# Genome Mining of Deep-Sea Cold Seep-Derived Fungus Reveals a Laccase–Fasciclin System Modulating Regioselective Naphthopyranone Dimerization

**DOI:** 10.3390/ijms27104156

**Published:** 2026-05-07

**Authors:** Hongcheng Li, Zhiting Li, Junpeng Sun, Xiaoyu Yang, Kaishuai Xing, Meixin Shi, Fei Xiao, Wenli Li

**Affiliations:** 1 Key Laboratory of Marine Drugs, Ministry of Education of China, School of Medicine and Pharmacy, Ocean University of China, Qingdao 266003, China; lihongcheng@stu.ouc.edu.cn (H.L.); sjp0625@126.com (J.S.); xingkaishuai566@126.com (K.X.); meixinshi126@163.com (M.S.); xiaofei3450@ouc.edu.cn (F.X.); 2 State Key Laboratory for Crop Stress Resistance and High-Efficiency Production, Shaanxi Key Laboratory of Natural Products & Chemical Biology, College of Chemistry & Pharmacy, Northwest A&F University, Yangling 712100, China; lizhiting@nwafu.edu.cn (Z.L.); yangxy@qdhhc.edu.cn (X.Y.); 3 Laboratory for Marine Drugs and Bioproducts, Qingdao Marine Science and Technology Center, Qingdao 266237, China

**Keywords:** deep-sea cold seep, naphthopyranones, genome mining, heterologous expression, laccase–fasciclin system

## Abstract

Naphthopyranones represent a structurally diverse family of fungal polyketides exhibiting a broad range of biological activities, including antibacterial, antifungal, and cytotoxic properties. Despite extensive investigations of terrestrial-derived naphthopyranones, the biosynthetic machinery responsible for their production in marine fungi has remained unexplored. Here, we report the first characterization of naphthopyranone biosynthetic gene clusters (BGCs) from a deep-sea-derived fungus. Genome mining of the cold seep-associated *Penicillium javanicum* OUCF108 revealed two highly homologous polyketide synthase gene clusters, *pig1* and *pig2*. Comparative transcriptomics combined with targeted disruption of the core PKS gene *pigA2* demonstrated that *pig2* is the essential BGC responsible for (*R*)-semivioxanthin (**1**) production. Stepwise reconstruction of the *pig2* pathway in *Aspergillus oryzae* NSAR1 unraveled the complete biosynthetic route from the heptaketide precursor *nor*-toralactone (**2**) to (*R*)-semivioxanthin (**1**) and its dimeric derivatives. In vitro biochemical characterization revealed that the *O*-methyltransferase PigN2 catalyzes regioselective 6-*O*-methylation with relaxed substrate specificity, that the laccase PigF2 mediates oxidative dimerization of **1** to afford dimeric derivatives, and that the fasciclin-like protein PigG2 alters this default regiochemistry, affording abundant alternative regioisomeric dimers alongside the 5,5′-linked product. Notably, a new naphthopyranone derivative, *nor*-4-hydroxy-toralactone (**4**), was isolated and structurally elucidated. Antimicrobial evaluation of all isolated compounds revealed that **4** exhibits moderate antifungal activity against the multidrug-resistant pathogen *Candida auris* (MIC = 12.5 μg mL^−1^). Structure–activity relationship analysis identified the C-4 hydroxyl moiety is critical for activity. This study highlights the potential of deep-sea fungi as an untapped reservoir of bioactive naphthopyranones and provides enzymatic insights for the construction of regioselectively coupled biaryl scaffolds.

## 1. Introduction

Deep-sea cold seeps constitute one of the most extreme habitats on Earth, characterized by high pressure, low temperature, permanent darkness, and low oxygen levels [1]. These harsh constraints have driven resident microorganisms to evolve distinctive metabolic pathways, often resulting in the production of secondary metabolites with unusual structural features and potent bioactivities [2,3]. Genome sequencing efforts have revealed that fungi harbor numerous biosynthetic gene clusters (BGCs), many of which remain orphan clusters with no characterized products [4,5]. Fungi inhabiting these extreme environments, in particular, represent an underexplored reservoir of biosynthetic potential [6]. Consequently, genome mining of these underexplored organisms has proven to be a productive strategy for discovering novel bioactive natural products [7,8,9].

Naphthopyranones are a class of fungal polyketides that display considerable structural diversity and a wide spectrum of pharmacological activities, including antibacterial, cytotoxic, and anticancer effects [10,11]. These compounds occur predominantly in filamentous fungi of the genera *Aspergillus*, *Fusarium*, *Penicillium*, and *Trichophyton*, though sporadic reports from higher plants also exist. To date, several naphthopyranone biosynthetic pathways have been elucidated [12,13,14,15,16,17,18,19,20,21,22]. The naphthopyranone scaffold is assembled by a non-reducing polyketide synthase (NR-PKS) through the iterative condensation of malonyl-CoA extender units, generating a heptaketide backbone that undergoes subsequent cyclization, aromatization, and release [23]. Downstream tailoring enzymes, including ketoreductases, *O*-methyltransferases, and oxidases, then modify the naphthopyranone core to afford structurally varied derivatives [13,14,16,17,18]. Bioinformatic analysis of naphthopyranone BGCs has revealed a conserved modular architecture centered on the NR-PKS, yet accompanied by considerable variation in the downstream tailoring gene complement, which accounts for the observed structural diversity within this compound family.

A characteristic biosynthetic feature of many naphthopyranone natural products is the oxidative dimerization of monomeric units to generate biaryl-coupled dimers, which frequently exhibit axial chirality arising from restricted rotation about the biaryl bond. Dimeric naphthopyranones such as vioxanthin, viomellein, xanthomegnin, and viriditoxin display broad biological activities, ranging from potent antibacterial effects against methicillin-resistant *Staphylococcus aureus* (MRSA) to antifungal, cytotoxic, and enzyme-inhibitory properties [10]. The dimerization reaction is catalyzed predominantly by laccases, multicopper oxidases that generate phenoxyl radicals capable of undergoing C–C bond formation [24]. However, laccases are generally regarded as nonspecific oxidases, leading to a mixture of isomeric products. To achieve precise regioselectivity, fungi are believed to employ auxiliary proteins including dirigent proteins and fasciclin domain-containing proteins, which template the radical coupling reaction [16,22,25]. For example, the viriditoxin system utilizes a laccase (Av-VilL) for dimerization, and fasciclin-like proteins are commonly encoded within naphthopyranone BGCs [26]. Despite their widespread occurrence, the exact molecular role of fasciclin domain proteins in governing laccase-mediated regioselective coupling remains poorly defined. Investigating this cooperative interplay is essential both for understanding how axially chiral dimers are assembled in nature and for harnessing these enzymes as biocatalysts.

In this study, we identified and functionally characterized two highly homologous naphthopyranone BGCs, *pig1* and *pig2*, from the genome of the deep-sea cold seep-derived fungus *Penicillium javanicum* OUCF108. By integrating comparative transcriptomics, targeted gene disruption in the native host, and systematic heterologous pathway reconstruction in *Aspergillus oryzae* NSAR1, we demonstrate that *pig2* is the essential BGC responsible for the biosynthesis of (*R*)-semivioxanthin (**1**) and its dimeric derivatives. Stepwise reconstitution of the *pig2* pathway revealed the complete biosynthetic logic from the heptaketide precursor to the final dimeric products. Moreover, in vitro biochemical characterization confirmed that the laccase PigF2 and the fasciclin-like protein PigG2 function as a cooperative enzymatic system that modulates the product profile of oxidative dimerization. Furthermore, the biosynthetic intermediate 7-*de*-*O*-methylsemivioxanthin (**4**) was found to exhibit potent antifungal activity against the globally emerging multidrug-resistant pathogen *Candida auris*. These findings highlight the critical regulatory role of fasciclin proteins in fungal polyketide biosynthesis and the value of deep-sea fungi as sources of bioactive natural products.

## 2. Results

### 2.1. Genome Mining Reveals Two Homologous Naphthopyranone PKS Gene Clusters in P. javanicum OUCF108

**Figure 1:**
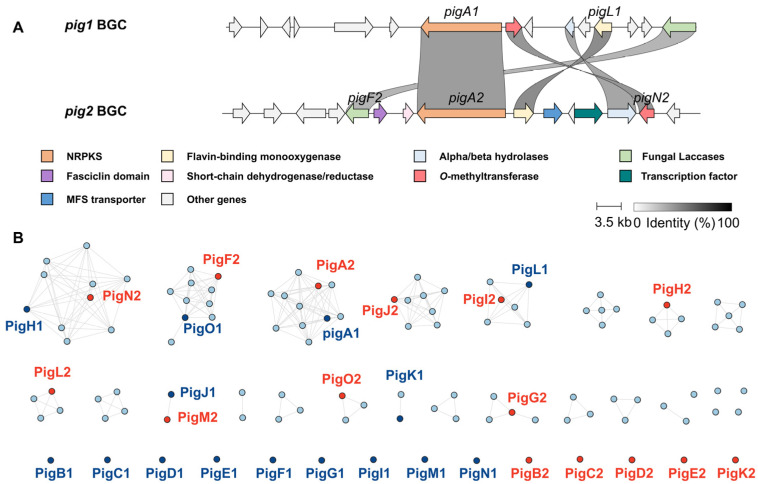
Composition and homology of the *pig1* and *pig2* gene clusters. (**A**) Schematic representation of the gene organization of the *pig1* and *pig2* loci. Genes are color-coded by predicted function. (**B**) Sequence Similarity Network (SSN) analysis of translated ORFs from *pig1*, *pig2*, and characterized naphthopyranone BGCs. Nodes represent individual protein sequences; edges connect sequences sharing significant similarity. Nodes are colored by cluster of origin: blue nodes represent proteins from the *pig1* BGC, and red nodes represent proteins from the *pig2* BGC.

The fungal strain OUCF108 was isolated from deep-sea cold seep sediments and identified as *P. javanicum* on the basis of its internal transcribed spacer (ITS) sequence (99.86% identity with the type strain). Whole-genome sequencing followed by bioinformatic analysis using antiSMASH detected 59 putative BGCs, of which 32 were classified as polyketide synthase (PKS) gene clusters, underscoring the extraordinary biosynthetic capacity of this deep-sea isolate. Among these, two highly homologous PKS loci attracted our attention: *pig1* (Cluster 2.2, 48.3 kb) and *pig2* (Cluster 39.1, 49.1 kb, Figure 1A). To evaluate the evolutionary relationships of these two clusters with respect to characterized naphthopyranone BGCs, we constructed a global Sequence Similarity Network (SSN) incorporating the translated open reading frames (ORFs) of *pig1*, *pig2*, and known naphthopyranone BGCs from public databases, including the vioxanthin and aurofusarin pathways (Figure 1B).

The SSN analysis revealed a pattern of conserved core biosynthetic logic coexisting with divergence in auxiliary functions. The core NR-PKS enzymes PigA1 (from *pig1*) and PigA2 (from *pig2*), which share 67% amino acid sequence identity, clustered tightly with established naphthopyranone synthesizing PKSs in the database, confirming their assignment as backbone-generating enzymes for naphthopyranone scaffolds. Similarly, several key tailoring enzymes from both clusters, including the putative *O*-methyltransferases (PigH1 and PigN2), the multicopper oxidases/laccases (PigO1 and PigF2), and the flavin-dependent monooxygenases (PigL1 and PigI2), also exhibited high homology with counterparts in known BGCs. While the core biosynthetic machinery is highly conserved, the SSN analysis highlighted the sequence-level divergence of these deep-sea-derived clusters by revealing unique auxiliary genes not commonly observed in previously characterized naphthopyranone BGCs. Specifically, isolated nodes encoding a glycosyltransferase (PigB1), an imidazoleglycerol–phosphate dehydratase (PigD1), a monooxygenase (PigG1) and a glycosyl hydrolase (PigC2) suggest the presence of potentially unexplored tailoring steps, such as potential glycosylation, in naphthopyranone biosynthesis. Moreover, *pig2* is characterized by the presence of a fasciclin domain-containing protein (PigG2) and a short-chain dehydrogenase (PigH2). These architectural differences suggest that *pig2* and *pig2* may possess a more diverse catalytic repertoire, particularly with respect to oxidative dimerization.

### 2.2. Comparative Transcriptomics and Targeted Gene Disruption Identify pig2 as the Essential Cluster for Naphthopyranone Biosynthesis

**Figure 2:**
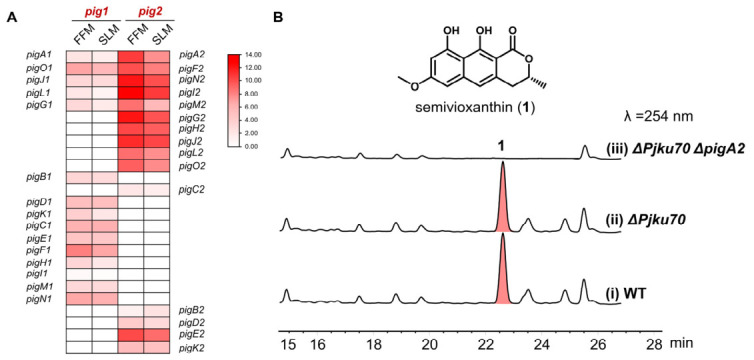
Comparative transcriptomics and targeted gene disruption establish *pig2* as the essential active naphthopyranone biosynthetic gene cluster. (**A**) Heatmap showing the transcriptional profiles of orthologous and unique genes within the *pig1* and *pig2* clusters under FFM and SLM culture conditions. The color scale represents the log_2_ (FPKM + 1) values, illustrating that *pig2* is actively transcribed whereas *pig1* remains largely silent. The blank cells indicate genes that are “not present” in the respective cluster. (**B**) Chemical structure of the dominant metabolite (*R*)-semivioxanthin (**1**) and HPLC analysis (*λ* = 254 nm) of culture extracts from the *P. javanicum* wild-type (WT), the NHEJ-deficient background strain (Δ*Pjku70*), and the *pigA2* disruption mutant (Δ*Pjku70*Δ*pigA2*).

The co-occurrence of two homologous naphthopyrone BGCs within a single genome raised the question of whether both clusters contribute to metabolite production. To address this, we performed comparative transcriptomic analysis under standard fermentation conditions that support naphthopyranone biosynthesis. RNA-seq data revealed that the core genes of *pig2*, including *pigA2* (NR-PKS), *pigN2* (*O*-methyltransferase), and *pigF2* (laccase), were expressed at high levels (FPKM > 600), whereas the corresponding orthologs in *pig1* (*pigA1*, *pigH1*, *pigO1*) expressed at very low levels (FPKM < 10) across all conditions tested (Figure 2A). These data indicated that *pig1* is transcriptionally silent under the conditions examined, while *pig2* is actively transcribed.

To establish a direct causal link between *pig2* and naphthopyranone production, we developed a genetic manipulation system for *P. javanicum* OUCF108. An NHEJ-deficient strain (Δ*ku70*) was first constructed to enhance homologous recombination efficiency. Subsequently, the core PKS gene *pigA2* was disrupted by homologous replacement in the Δ*ku70* background. HPLC analysis of the Δ*pigA2* mutant culture extracts showed complete abolishment of (*R*)-semivioxanthin (**1**) production, whereas the wild-type strain accumulated **1** as the dominant metabolite (Figure 2B). No compensatory production of naphthopyranones was observed in the Δ*pigA2* mutant, confirming that *pig1* cannot substitute for *pig2* even when the pathway is inactivated. These results established that under the tested conditions, *pig2* is the essential BGC responsible for naphthopyranone biosynthesis, and *pig1* cannot compensate for the loss of *pigA2*.

### 2.3. Heterologous Expression of pig1 and pig2 BGC in A. oryzae NSAR1

To further validate the functional status of the two clusters and to enable stepwise pathway dissection, we pursued heterologous expression of both *pig1* and *pig2* in the well-established fungal host *A. oryzae* NSAR1.

**Figure 3:**
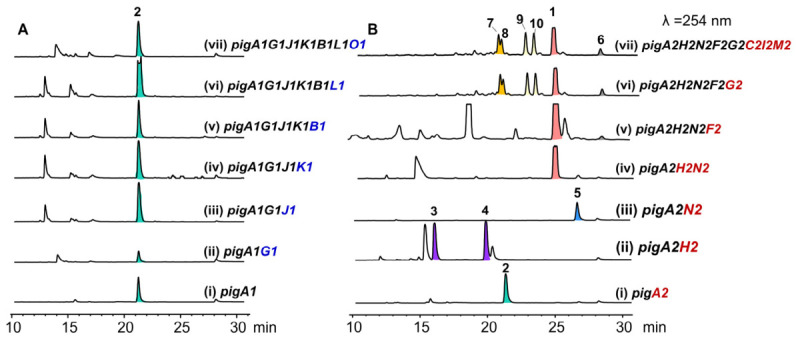
HPLC profiles of *A. oryzae* NSAR1 transformants. (**A**) *pig1* BGC; (**B**) *pig2* BGC. Peaks labeled **1**–**10** correspond to the compounds described in the text, with colors for visual distinction.

We began by introducing the *pig2* core PKS gene *pigA2* alone into *A. oryzae* NSAR1. HPLC analysis of the transformant culture extracts revealed a new metabolite peak **2** absent from the empty-vector control (Figure 3B). This result confirmed PigA2 as a heptaketide synthase that catalyzes the iterative condensation of one acetyl-CoA starter unit with six malonyl-CoA extender units, followed by regioselective cyclization and aromatization to afford the naphthopyrone scaffold. To identify the downstream tailoring steps, we next co-expressed *pigA2* with the short-chain dehydrogenase gene *pigH2* and the *O*-methyltransferase gene *pigN2*. The resulting transformant (AO-*pigA2H2N2*) produced a new major peak (**1**), which was identified as (*R*)-semivioxanthin by HR-ESI-MS comparison with an authentic standard (Figure 3B). This result demonstrated that PigH2 and PigN2 together are sufficient to convert **2** to (*R*)-semivioxanthin (**1**), establishing their involvement in the semivioxanthin biosynthetic pathway. To elucidate the individual contributions of PigH2 and PigN2, we performed independent co-expression experiments. When *pigA2* was co-expressed with *pigH2* alone (AO-*pigA2H2*), two new metabolite peaks (**3** and **4**) were observed in addition to **2** (Figure 3B). When *pigA2* was co-expressed with *pigN2* alone (AO-*pigA2N2*), a single new peak **5** was detected (Figure 3B). These results indicated that PigH2 and PigN2 catalyze distinct modifications on the scaffold **2**, and that the combined action of both enzymes channels the intermediates toward (*R*)-semivioxanthin (**1**).

Bioinformatic analysis revealed that the gene cluster encodes a laccase (PigF2) and a fasciclin domain-containing protein (PigG2), both of which are potentially implicated in the dimerization process. Previous reports have demonstrated that laccases often require fasciclin domain-containing partners to efficiently catalyze oxidative dimerization reactions [21,25]. To dissect the individual catalytic functions and synergistic mechanism of PigF2 and PigG2, we first introduced the laccase gene *pigF2* into the AO-*pigA2H2N2* background to examine whether PigF2 alone is sufficient for dimerization. The transformant AO-*pigA2H2N2F2* accumulated new peaks whose molecular masses, as determined by HR-ESI-MS, were consistent with homodimeric naphthopyrones (Figure 3B). These data indicated that PigF2 catalyzes the oxidative dimerization of (*R*)-semivioxanthin (**1**). Subsequently, to explore the regulatory role of PigG2, we co-introduced *pigF2* and *pigG2*. The resulting transformant AO-*pigA2H2N2F2G2* produced a distinct set of dimeric peaks that differed from those generated by AO-*pigA2H2N2F2* (Figure 3B), indicating that PigG2 redirects the regiochemistry of the laccase-mediated coupling reaction. Notably, alongside the alternative regioisomeric dimers **7** and **8** (MW = 546), two additional minor peaks, **9** and **10**, were detected in the AO-*pigA2H2N2F2G2* extract. LC-MS analysis revealed that **9** and **10** possess a molecular weight of 548, suggesting they are hydrogenated products of the dimeric species. Introduction of the remaining genes putatively encoding glycosyl hydrolase PigC2, flavin-dependent monooxygenase *pigI2* and serine hydrolase *pigM2*, did not yield any new products.

To independently evaluate the functionality of *pig1*, we performed a parallel series of heterologous expression experiments. Introduction of the *pig1* core PKS gene *pigA1* alone into *A. oryzae* NSAR1 (AO-*pigA1*) afforded **2** as the product, identical to that generated by *pigA2* (Figure 3A, panel i). This result demonstrated that PigA1 retains a functional polyketide synthase activity capable of assembling the same heptaketide backbone as PigA2. However, when we co-expressed *pigA1* with the remaining *pig1* tailoring genes, no new metabolite peaks were detected beyond **2** (Figure 3A, panels ii~vii). These results indicated that although the *pig1* PKS gene pigA1 encodes a functional backbone generating enzyme, the downstream tailoring genes within *pig1* are non-functional in the heterologous host. Taken together with the transcriptomic and gene disruption data, these findings confirmed that *pig1* has lost its capacity for complete pathway execution, while *pig2* constitutes the essential naphthopyranone BGC in *P. javanicum* OUCF108.

### 2.4. Structural Elucidation of **1**–**5**

To assign the structures of all metabolites generated during heterologous expression, compounds **1**–**5** were isolated by a combination of silica gel column chromatography, ODS column chromatography and semi-preparative HPLC. Their structures were elucidated by HR-ESI-MS, 1D NMR (^1^H, ^13^C, DEPT), and 2D NMR (^1^H–^1^H COSY, HSQC, HMBC) spectroscopy.

Compounds **1**, **2**, **3**, and **5** were identified as (*R*)-semivioxanthin [27], *nor*-toralactone [23], 7-*de*-*O*-methylsemivioxanthin [28], and toralactone [29], respectively, by comparison of their spectroscopic data with those reported in the literature.

**Figure 4:**
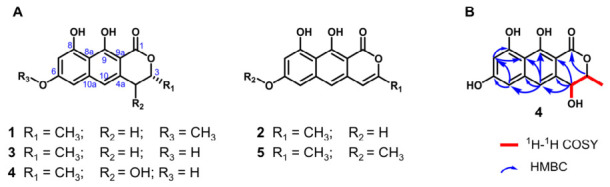
(**A**) Structures of compounds **1**–**5**; (**B**) Key 2D NMR correlations (^1^H–^1^H COSY and HMBC) of compound **4**.

Compound **4** was assigned a molecular formula of C_14_H_12_O_5_ on the basis of its HR-ESI-MS data ([M + H]^+^ at *m*/*z* 277.0800, calcd 277.0712). The ^1^H and ^13^C NMR spectra of **4** closely resembled those of *nor*-toralactone (**2**); however, the appearance of two methine signals at *δ*_H_ 4.70 (H-3) and 4.51 (H-4) indicated a structural modification within the *γ*-pyrone ring (Table 1). ^1^H–^1^H COSY correlations of 3-CH_3_/H-3/H-4 established the connectivity of the C-3–C-4 fragment. Key HMBC correlations from H-4 to C-4a, C-9a, and C-10; from H-10 to C-5, C-8, C-8a, C-9, C-9a, and C-10a; from H-5 to C-6 and C-7; and from H-7 to C-8 confirmed the placement of the hydroxyl group at C-4 (Figure 4). On the basis of these data, **4** was established as a new naphthopyranone derivative bearing a 4-hydroxyl substituent on the pyrone ring, and was named *nor*-4-hydroxy-toralactone.

To determine the absolute configuration of **4**, we combined biosynthetic logic with quantum chemical calculations. The absolute configuration at C-3 in the final biosynthetic product (*R*)-semivioxanthin (**1**) has been previously established as *R* [27]. Given that **4** shares the same biosynthetic machinery and precursor (**3**) with **1**, the stereocenter at C-3 in **4** is biosynthetically conserved and thus deduced to be *R*. For the C-4 stereocenter, the experimental electronic circular dichroism (ECD) spectrum of **4** was recorded and compared with the time-dependent density functional theory (TDDFT)-calculated ECD spectra of the two possible epimers, (4*S*)-**4** and (4*R*)-**4**. The calculated ECD curve of the (4*R*) isomer match with the experimental data (Figure S12). Consequently, the absolute configuration of **4** was assigned as 3*R*, 4*R*.

All dimeric products proved to be unstable under the isolation conditions, precluding their purification in sufficient quantity for full NMR characterization. Nonetheless, the structures of these products were tentatively assigned on the basis of HR-ESI-MS data and comparison with literature precedents [27]. The [M + H]^+^ ions of the major dimeric peaks (**6**/**7**/**8**) were observed at *m*/*z* 546.1523, consistent with the molecular formula C_30_H_22_O_10_ and the mass change expected for oxidative homodimerization of (*R*)-semivioxanthin (**1**) (2M − 2H). Furthermore, the newly emerged peaks **9** and **10** exhibited [M + H]^+^ ions at *m*/*z* 549.1755, corresponding to a molecular formula of C_30_H_24_O_10_. This 2 Da mass increase relative to **7** and **8** indicates that **9** and **10** are hydrogenated dimeric byproducts, a phenomenon frequently observed when highly reactive radical or quinonoid intermediates are quenched during laccase-mediated coupling. The UV absorption spectrum of **6** (Figures S13 and S14) matched that reported for pigmentosin A [27], leading to its tentative identification as pigmentosin A, a 5,5′-linked homodimer. Although the inherent instability of compounds **7**–**10** prevented their complete structural characterization by NMR, their distinct HPLC retention times and UV absorption profiles strongly indicate that **7** and **8** are alternative regioisomers or atropisomers differing from the 5,5′-linked dimer **6** (Figures S15–S22).

### 2.5. In Vitro Biochemical Characterization of PigN2, PigF2, and PigG2

**Figure 5:**
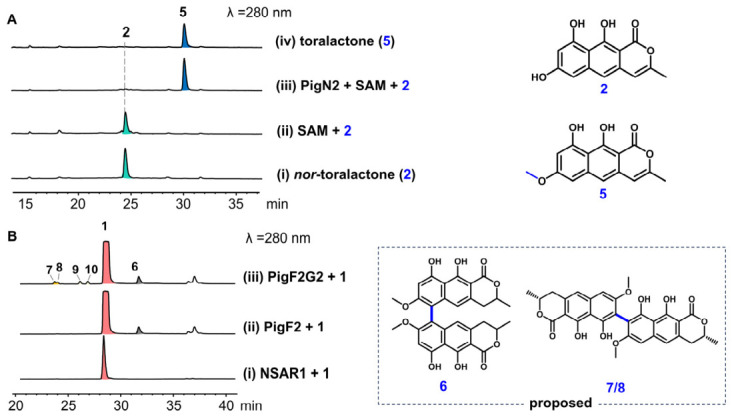
In vitro biochemical characterization of PigN2 and PigF2/G2. (**A**) HPLC traces showing the conversion of *nor*-toralactone (**2**) to toralactone (**5**) by purified PigN2 in the presence of SAM. (**B**) HPLC analysis of the conversion of (*R*)-semivioxanthin (**1**) with PigF2/PigG2-containing lysate obtained from *A. oryzae* coexpression cultures.

To further characterize the catalytic properties of the key tailoring enzymes, we performed in vitro biochemical assays with purified recombinant PigN2, PigF2, and PigG2. Incubation of PigN2 with *nor*-toralactone (**2**) and SAM afforded a single product identified as toralactone (**5**) by HPLC and HR-ESI-MS, confirming regioselective 6-*O*-methylation (Figure 5A, panel iii). To explore substrate scope of PigN2, the emodin, DHN, and YWA3 were evaluated as alternative substrates. HPLC analysis showed that PigN2 completely converted the structural analog *nor*-rubrofusarin to rubrofusarin and partially transformed emodin to physcion. In contrast, no catalytic activity was observed toward DHN, a small-molecule substrate with a markedly distinct structure (Figure S4). These results demonstrate that the *O*-methyltransferase PigN2 possesses a degree of substrate flexibility, yet its catalytic activity is strictly biased toward compounds bearing a polycyclic aromatic scaffold analogous to its native substrate.

To directly verify the cooperative catalytic function of the laccase PigF2 and the fasciclin protein PigG2 in vitro, we sought to reconstitute their activity in a cell-free system. However, in vitro reconstitution of laccase–fasciclin complexes is notoriously challenging: heterologously purified laccases frequently lose activity due to protein misfolding, loss of essential copper cofactors, or failure to assemble a stable functional complex with their fasciclin partners. Drawing on these precedents, we adopted a fungal cell-free lysate strategy using co-expression transformant AO-*pigF2G2* [15,16,18,19,25,26]. HPLC analysis of the reaction mixture revealed the formation of the 5,5′-linked dimer **6** when PigF2 was added (Figure 5B, panel ii). Strikingly, the addition of PigG2 to the reaction led to the accumulation of additional regioisomeric dimers **7** and **8**, along with the hydrogenated byproducts **9** and **10** (Figure 5B, panel iii). This in vitro reconstitution mirrors the in vivo heterologous expression profile, confirming the cooperative role of the PigF2–PigG2 complex. As a control, cell-free lysate prepared from *A. oryzae* NSAR1 did not generate any dimeric products when incubated with **1** under identical conditions (Figure 5B, panel i), confirming that the observed dimerization activity is attributable to the co-expressed PigF2–PigG2 complex.

These cell-free lysate results were fully consistent with the in vivo heterologous expression data from AO-*pigA2H2N2F2G2*, providing independent biochemical evidence that PigF2 and PigG2 function as a cooperative enzymatic system to catalyze the oxidative dimerization of (*R*)-semivioxanthin (**1**). The requirement for co-expression within the same cell further supports a model in which PigG2 physically associates with PigF2 during or shortly after translation, forming a complex that redirects the regiochemistry of the laccase-generated radical intermediates away from the default 5,5′-coupling and toward alternative C–C bond formation. Together, the in vivo and in vitro data established the laccase PigF2 and the fasciclin protein PigG2 as the molecular determinants governing regioselective naphthopyrone dimerization in the *pig2* pathway.

### 2.6. Antimicrobial Activity of Isolated Compounds

The antimicrobial activities of compounds **1**–**5** were evaluated against a panel of multidrug-resistant (MDR) bacteria and pathogenic fungi using the broth microdilution method (Table S7). Compound **2** (*nor*-toralactone) exhibited moderate antibacterial activity against *Staphylococcus aureus* CCARM 3090 with a minimum inhibitory concentration (MIC) of 12.5 μg mL^−1^. The new derivative *nor*-4-hydroxy-toralactone (**4**) displayed moderate antifungal activity against *C. auris* with an MIC of 12.5 μg mL^−1^. All other compounds exhibited no detectable antimicrobial activity at concentrations up to 50 μg mL^−1^.

Structure–activity relationship (SAR) analysis of the bioactivity data revealed instructive trends. Comparison of *nor*-toralactone (**2**, active against *S. aureus*) with its methylated derivative toralactone (**5**, inactive) indicated that the free hydroxyl group at C-6 is critical for antibacterial activity. Furthermore, comparison between compound **4** (active against *C. auris*) and compound **2** (inactive against *C. auris*) revealed that they differ only by the presence of a hydroxyl group at C-4. This confirms that the C-4 hydroxylation is a critical moiety for antifungal activity against *C. auris*. These SAR insights suggest that specific hydroxylations on the naphthopyranone scaffold dictate target selectivity, providing valuable structural guidelines for future optimization.

### 2.7. Proposed Biosynthetic Pathway

**Figure 6:**
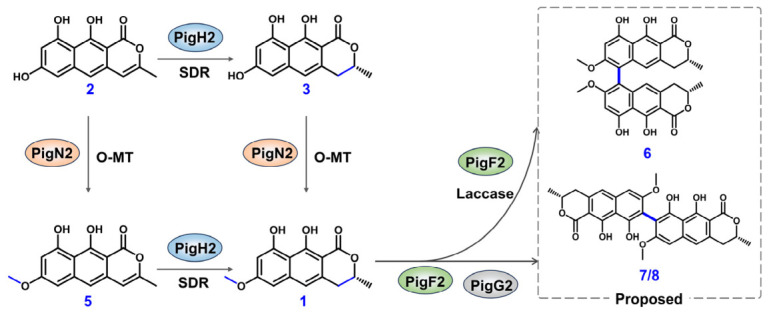
Proposed biosynthetic pathway for (*R*)-semivioxanthin and its dimeric derivatives from *P. javanicum OUCF108*.

Based on the collective results of heterologous reconstitution and biochemical characterization, we propose the following biosynthetic pathway for (*R*)-semivioxanthin and its dimeric derivatives (Figure 6). The biosynthesis is initiated by the non-reducing polyketide synthase PigA2, which catalyzes the assembly of the heptaketide backbone *nor*-toralactone (**2**) from one molecule of acetyl-CoA and six molecules of malonyl-CoA. The short-chain dehydrogenase/reductase PigH2 then reduces the C-7 carbonyl group of **2** to a hydroxyl group, yielding 7-*de*-*O*-methylsemivioxanthin (**3**). The *O*-methyltransferase PigN2 subsequently transfers a methyl group from SAM to the 6-OH of **3**, producing (*R*)-semivioxanthin (**1**). Alternatively, PigN2 can methylate *nor*-toralactone (**2**) directly to afford toralactone (**5**), which is subsequently reduced by PigH2 to also yield **1**, demonstrating a flexible tailoring order.

The laccase PigF2 then catalyzes the oxidative radical coupling of (*R*)-semivioxanthin (**1**). In the absence of PigG2, PigF2 generates predominantly the 5,5′-linked dimer **6**. In the presence of the fasciclin-like protein PigG2, the regioselectivity of the coupling reaction is significantly altered, generating the alternative regioisomeric dimers **7** and **8**, as well as their hydrogenated byproducts **9** and **10**, alongside the default dimer **6**. Finally, the flavin-dependent monooxygenase PigI2 oxidizes monomeric or dimeric intermediates to generate semixanthomegnin (**10**) or other oxidized derivatives.

## 3. Discussion

The co-occurrence of two highly homologous naphthopyranone BGCs within the genome of *P. javanicum* OUCF108 afforded a rare opportunity to investigate the evolutionary dynamics of duplicated biosynthetic loci. Through an integrated approach combining comparative transcriptomics, targeted gene disruption, and heterologous pathway reconstruction, we established that *pig2* is the primary active cluster responsible for the biosynthesis of (*R*)-semivioxanthin (**1**) and its dimeric derivatives, while *pig1* is expressed at a very low level and functionally inert intracellularly. Notably, the core NR-PKS PigA1 within *pig1* retains full catalytic competence, assembling the same heptaketide scaffold *nor*-toralactone (**2**) as its *pig2* counterpart PigA2. However, the downstream tailoring enzymes within *pig1* failed to convert **2** to any further products in the heterologous host. These data point to *pig1* as an evolutionary pseudo-gene cluster in which the accumulation of mutations has abolished the auxiliary tailoring functionalities while the core backbone synthesizing capacity remains intact. Such genomic redundancy followed by asymmetric functional decay is consistent with a model in which duplicated BGCs undergo divergent selection: the active copy (*pig2*) is maintained under purifying selection, while the redundant copy (*pig1*) drifts toward pseudogenization. This pattern underscores the importance of experimental validation when assigning biosynthetic function to paralogous gene clusters identified through genome mining, as sequence homology alone is insufficient to predict functional competence [30].

Stepwise reconstitution of the *pig2* pathway in *A. oryzae* NSAR1 revealed the complete biosynthetic logic from *nor*-toralactone (**2**) to the functionalized monomer (*R*)-semivioxanthin (**1**). The *O*-methyltransferase PigN2 and the short-chain dehydrogenase PigH2 act on **2** through parallel yet convergent modifications that are channeled toward **1** when both enzymes operate in concert. In vitro biochemical characterization demonstrated that PigN2 catalyzes regioselective 6-*O*-methylation of **2** to afford toralactone **5**, with no detectable methylation at C-7, C-8, or C-10. Beyond its native substrate, PigN2 displayed relaxed substrate specificity, completely converting the structural analog *nor*-rubrofusarin to rubrofusarin and partially transforming emodin to physcion, while showing no activity toward smaller substrates such as DHN that lack the extended polycyclic aromatic framework. This substrate scope profile indicates that PigN2 recognizes the overall topology of the naphthopyranone scaffold rather than a single functional group, a property that positions PigN2 as a versatile biocatalytic tool for the regioselective *O*-methylation of diverse polycyclic aromatic natural products.

A central finding of this study is the functional characterization of the cooperative interplay between the laccase PigF2 and the fasciclin-like protein PigG2 during oxidative dimerization. Laccases are generally regarded as nonspecific oxidases that generate phenoxyl radicals without inherent control over the site of C–C bond formation [15]. Consistent with this view, heterologous expression of PigF2 alone (AO-*pigA2H2N2F2*) and the corresponding cell-free lysate assay both produced the 5,5′-linked dimer pigmentosin A (**6**) as the default product. Co-expression of PigG2 with PigF2, either in vivo (AO-*pigA2H2N2F2G2*) or in the cell-free lysate system, altered the regiochemical outcome, affording the additional regioisomeric dimers **7** and **8** alongside **6**. The observation that a catalytically competent PigF2–PigG2 complex can only be obtained through co-expression within the same cell, and not by mixing individually expressed lysates, indicates that PigG2 must physically associate with PigF2 during or shortly after translation to form a functional unit. We propose that the fasciclin domain serves as a structural scaffold that physically engages the laccase active site, reorienting the radical intermediates to facilitate alternative C–C bond formations, thereby expanding the product profile beyond the default 5,5′-coupling [25]. The formation of the hydrogenated byproducts **9** and **10** during the PigG2-directed coupling further supports a radical-based mechanism. We postulate that the fasciclin protein PigG2 not only provides a steric template to prevent the default 5,5′-coupling but also stabilizes specific radical or quinone methide intermediates, a fraction of which undergoes off-target reduction to yield **9** and **10**. The laccase–fasciclin paradigm thus represents a general enzymatic strategy deployed by fungi to generate regioselectively coupled biaryl scaffolds from common monomeric precursors.

The antimicrobial evaluation of the isolated naphthopyranones revealed instructive SAR centered on the C-6 methylation state. *nor*-toralactone (**2**), which bears a free hydroxyl at C-6, exhibited moderate antibacterial activity against *S. aureus* CCARM 3090 (MIC = 12.5 μg mL^−1^), whereas its 6-*O*-methylated derivative toralactone (**5**) was inactive (MIC > 50 μg mL^−1^). An analogous pattern was observed for antifungal activity against *C. auris*: the newly identified derivative *nor*-4-hydroxy-toralactone (**4**), possessing a free 4-OH, displayed an MIC of 12.5 μg mL^−1^. Comparative SAR analysis revealed that the absence of the C-4 hydroxyl group completely abolishes the antifungal activity, as evidenced by the inactivity of **2**. These results indicate that the C-4 hydroxyl moiety is critical for activity, likely interacting with specific molecular targets via hydrogen bonding. This SAR insight provides a structural starting point for the future semi-synthetic optimization of naphthopyranone-based antifungal agents.

## 4. Materials and Methods

### 4.1. General Experimental Procedures

1D and 2D NMR spectra were acquired on Bruker Avance III 400, 500, and 600 MHz spectrometers (Bruker BioSpin, Rheinstetten, Germany). Chemical shifts are reported in δ (ppm), referenced to the residual solvent peaks (δ_H_ 2.50 and δ_C_ 39.52 for DMSO-*d*_6_). High-resolution electrospray ionization mass spectrometry (HR-ESI-MS) data were recorded on a Thermo Scientific LTQ Orbitrap XL spectrometer (Thermo Fisher Scientific, Waltham, MA, USA). HPLC analysis was performed on an Agilent 1260 Infinity II system (Agilent Technologies, Santa Clara, CA, USA) equipped with a diode array detector (DAD) using a YMC Triart C18 column (5 μm, 4.6 × 250 mm, YMC Co., Ltd., Kyoto, Japan). Column chromatography utilized silica gel (Qingdao Haiyang Chemical Co., Ltd., Qingdao, China) and ODS (40 μm, 120 Å, Jinan Bona Biological Technology, Jinan, China).

### 4.2. Bioinformatic Analysis

Sequence similarity network analysis was performed using the EFI-EST web server (https://efi.igb.illinois.edu/efi-est/, accessed on 10 January 2025). Sequence similarity network (SSN) visualization was performed using Cytoscape v3.10.4 (The Cytoscape Consortium, San Diego, CA, USA). Fungal biosynthetic gene cluster (BGC) prediction was performed using antiSMASH fungal version v7.0 (https://fungismash.secondarymetabolites.org/, accessed on 23 May 2023).

### 4.3. Strains, Media, and Culture Conditions

The deep-sea-derived fungus *P. javanicum* OUCF108 was preserved in glycerol stocks at −80 °C. *A. oryzae* NSAR1, kindly provided by Prof. Ikuro Abe (The University of Tokyo), served as the heterologous expression host. *E. coli* DH5α was used for general plasmid construction and *E. coli* BL21(DE3) for recombinant protein expression. *E. coli* strains were grown in LB medium at 37 °C; fungal strains were typically cultured at 30 °C. *P. javanicum* OUCF108 was fermented in FLM3 medium for metabolite production; *A. oryzae* strains were cultured in DPY medium for transformation. Antibiotics were added where appropriate: ampicillin (100 μg mL^−1^) or kanamycin (50 μg mL^−1^) for *E. coli*; G418 (400 μg mL^−1^) and bleomycin (100 μg mL^−1^) for *P. javanicum*; pyrithiamine (0.25 μg mL^−1^) for *A. oryzae*.

### 4.4. Genetic Transformation of P. javanicum OUCF108

Protoplast preparation and PEG-mediated transformation of *P. javanicum* OUCF108 were performed following a modified protocol [30]. Briefly, spores were inoculated into 50 mL PDB medium and cultured at 30 °C, 220 rpm for 14–16 h. Harvested mycelia were mechanically fragmented using a FastPrep-24 5G instrument (MP Biomedicals, Irvine, CA, USA, 6500 rpm, 30 s), regenerated in fresh PDB (30 °C, 220 rpm, 3 h), and enzymatically digested with 1% snailase, 1% lywallzyme, and 1% cellulase (from *A. niger*) in 1.2 M MgSO_4_ at 30 °C, 130 rpm for 2.5 h. Protoplasts were purified by filtration and trapping buffer centrifugation. For gene disruption, approximately 1.2 kb upstream and downstream homologous arms flanking *ku70* or *pigA2* were amplified from genomic DNA and fused to bleomycin (*ble*) or G418 (*neo*) resistance cassettes by fusion PCR (Figure S1). Resulting constructs were transformed into protoplasts, and transformants were selected on regeneration medium containing appropriate antibiotics and verified by diagnostic PCR.

### 4.5. Construction of Fungal Expression Plasmids

Genes from the *pig2* cluster (*pigA2*, *pigH2*, *pigN2*, *pigF2*, *pigG2*, *pigI2*, *pigC2*, and *pigM2*) were amplified from *P. javanicum* OUCF108 genomic DNA using primers listed in Table S4. Each fragment was cloned into linearized pTAex3 or pUSA vectors using a Seamless Assembly Cloning Kit (Taihe Biotechnology, Beijing, China). Multi-gene expression plasmids were constructed by sequentially ligating *amyB* promoter (P*_amyB_*)–target gene–*amyB* terminator (T*_amyB_*) cassettes into the restriction sites of pUSA, pAdeA, or pPTRI destination vectors. All constructs were verified by DNA sequencing and list in Table S5.

### 4.6. Heterologous Expression in A. oryzae NSAR1

Transformation of *A. oryzae* NSAR1 was performed by the protoplast-PEG method [31]. Mycelia were digested in a solution containing 1% snailase, 1% lywallzyme, 1% cellulase, and 7.9% (NH_4_)_2_SO_4_ in 0.58% maleic acid. Transformants were selected on minimal medium lacking specific nutrients (arginine, methionine, or adenine) or containing pyrithiamine (0.25 μg mL^−1^), depending on the selectable markers. A complete list of transformant strains is provided in Table S6.

### 4.7. Analysis, Isolation, and Purification of Compounds

For metabolite analysis, *A. oryzae* transformants were cultured on DPY solid medium at 30 °C for 3 days; *P. javanicum* strains were cultured in FLM3 liquid medium at 30 °C for 7 days. Cultures were extracted with EtOAc, concentrated, dissolved in MeOH, and analyzed by HPLC using a linear gradient from 5% to 100% B/A in 35 min (A: H_2_O + 0.05% TFA; B: MeCN + 0.05% TFA; flow rate: 1.0 mL min^−1^).

For compound isolation, large-scale fermentations were performed (1 L DPY for *A. oryzae*; 5 L FLM3 for *P. javanicum*). Crude extracts were fractionated by silica gel and ODS column chromatography, followed by semi-preparative HPLC (YMC-Pack ODS-A, YMC Co., Ltd., Kyoto, Japan; flow rate: 2.0 mL min^−1^) to afford compounds **1**–**5**.

### 4.8. ECD Measurement and Computational Details

The experimental ECD spectrum of **4** was recorded on a JASCO J-1500 circular dichroism spectrometer (JASCO Corporation, Tokyo, Japan) in MeOH. For the computational analysis, conformational searches of the (4*S*) and (4*R*) isomers of **4** were performed using Crest v2.12 (developed by the Grimme group, University of Bonn, Bonn, Germany) at the GFNFF level of theory, followed by pre-optimization at the GFN2-xTB level (using the xtb program v6.6.1, developed by the Grimme Group, University of Bonn, Bonn, Germany) with a 4 kcal/mol energy window to remove high-energy conformers [32,33]. Geometry optimization and frequency calculations for each selected conformer were performed using the ORCA v5.0.3 (developed by the Neese Group, Max Planck Institute for Coal Research, Mülheim an der Ruhr, Germany) at the B3LYP/6-31G(d,p) level of theory with the CPCM polarizable conductor calculation model in MeOH. The theoretical ECD spectra for the optimized conformers were calculated using the Time-Dependent Density Functional Theory (TDDFT) method at the B3LYP/6-31G(d,p)level. The theoretical ECD spectra for the optimized conformers were then calculated using the Time-Dependent Density Functional Theory (TDDFT) method at the B3LYP/6-31G(d,p) level, also using ORCA v5.0.3. The final calculated ECD spectra were generated and plotted using the Multiwfn v3.8 software (developed by Tian Lu, Beijing Kein Research Center for Natural Sciences, Beijing, China) by applying a Lorentzian band shape with a half-bandwidth (sigma) of 1 eV [34,35]. The calculated spectra were scaled and compared directly with the experimental data.

### 4.9. Protein Expression and In Vitro Enzymatic Assays

For recombinant expression of PigN2, the pET28a-PigN2 plasmid was transformed into *E. coli* BL21(DE3). Cultures were grown in LB with kanamycin (50 μg mL^−1^) at 37 °C to OD_600_ = 0.6, induced with 0.1 mM IPTG, and incubated at 16 °C overnight. His_6_-tagged PigN2 was purified by Ni-NTA chromatography (Qiagen, Hilden, Germany). For the in vitro methyltransferase assay, the reaction mixture (100 μL) contained 100 mM Tris-HCl (pH 8.0), 200 μM SAM, 100 μM *nor*-toralactone (**2**), and 10 μM purified PigN2, and was incubated at 30 °C for 4 h prior to quenching with MeOH and HPLC analysis.

For characterization of the laccase PigF2 and the fasciclin-like protein PigG2, a cell-free lysate system was employed. Mycelia of relevant *A. oryzae* transformants were frozen in liquid nitrogen, ground to powder, resuspended in 50 mM Tris-HCl buffer (pH 8.0), and centrifuged (12,000× *g*, 20 min, 4 °C). (*R*)-semivioxanthin (**1**, 0.1 mM) was added to the supernatant and incubated at 30 °C for 4 h. Products were extracted with EtOAc and analyzed by HPLC-MS.

### 4.10. Antimicrobial Activity Assay

MIC values were determined by the broth microdilution method against a panel of MDR bacteria and pathogenic fungi. Bacterial strains (*S. aureus* CCARM 3090, *Enterococcus faecalis* CCARM 5172, *Enterococcus faecium* CCARM 5203, *Micrococcus luteus* ML01, *E. coli* CCARM 1009, *Salmonella typhimurium* CCARM 8250, *Acinetobacter baumannii* ATCC 19606, *Klebsiella pneumoniae* ATCC 13883, and *Pseudomonas aeruginosa* 15690) were cultured in LB broth at 37 °C. Fungal strains (*Candida albicans* CMCC(F) 98001 and *C. auris*) were cultured in PDB at 30 °C. Compounds were serially diluted (0.1–50 μg mL^−1^) in 96-well plates, inoculated with microbial suspensions (10^6^ CFU mL^−1^), and incubated for 24 h. Absorbance at 600 nm was measured on a BioTek Epoch 2 microplate spectrophotometer. Tetracycline and amphotericin B served as positive controls for bacteria and fungi, respectively.

## 5. Conclusions

This study elucidated the complete biosynthetic pathway for (*R*)-semivioxanthin (**1**) and its dimeric derivatives in the deep-sea cold seep-derived fungus *P. javanicum* OUCF108. Through genome mining, comparative transcriptomics, targeted gene disruption, and heterologous pathway reconstruction in *A. oryzae* NSAR1, we established that *pig2* is the essential naphthopyranone BGC, while *pig1* represents an evolutionary relic in which the core PKS remains functional but the downstream tailoring enzymes have lost activity.

The laccase PigF2 and the fasciclin-like protein PigG2 were shown to operate as a cooperative enzymatic system that governs the regioselectivity of oxidative dimerization. PigG2 alters the regioselectivity of the PigF2-catalyzed reaction, generating additional regioisomeric dimers (**7** and **8**) alongside the default 5,5′-coupled product. This work provides one of the first functional demonstrations that a single fasciclin domain protein can effectively modulate the product landscape of laccase-mediated biaryl coupling, expanding the enzymatic toolbox available for constructing axially chiral natural product scaffolds.

The biosynthetic intermediate *nor*-4-hydroxy-toralactone (**4**) displayed moderate antifungal activity against the multidrug-resistant pathogen *C. auris* (MIC = 12.5 μg mL^−1^). SAR analysis identified the C-4 hydroxyl moiety as an essential pharmacophore. Collectively, this study underscores the significance of exploring deep-sea fungi as an untapped reservoir for bioactive naphthopyranones, providing a solid structural starting point for the future semi-synthetic optimization of antimicrobial agents and the biocatalytic engineering of complex biaryl scaffolds.

## Figures and Tables

**Table 1:** NMR data for **4** in DMSO-*d*_6_ (400 and 150 MHz).

Position	*δ*_C_	*δ*_H_ (*J* in Hz)
1	160.6, C=O	
2		
3	152.2, C	
4	103.5, CH	6.24 (1H, d, *J* = 0.5 Hz)
4a	135.1, C	
5	98.0, CH	6.11 (1H, d, *J* = 1.9 Hz)
6	162.4, C	
7	96.3, CH	6.59 (1H, d, *J* = 1.9 Hz)
8	158.7, C	
8a	110.1, C	
9	157.2, C	

## Data Availability

The original contributions presented in this study are included in the article/Supplementary Materials. Further inquiries can be directed to the corresponding author.

## Supplementary Materials

The following supporting information can be downloaded at: https://www.mdpi.com/article/10.3390/ijms27104156/s1.
