# Supplementary material for: Genome Mining of Deep-Sea Cold Seep-Derived Fungus Reveals a Laccase–Fasciclin System Modulating Regioselective Naphthopyranone Dimerization
Source: Int J Mol Sci. 2026 May 7;27(10):4156. doi: 10.3390/ijms27104156 (PMC13207265; doi:10.3390/ijms27104156)

Supporting Information

Genome Mining of Deep-Sea Cold Seep-Derived Fungus Reveals a Laccase–Fasciclin System Modulating Regioselective Naphthopyranone Dimerization

Hongcheng Li [1], Zhiting Li [3], Junpeng Sun [1], Xiaoyu Yang [3], Kaishuai Xing [1], Meixin Shi [1], Fei Xiao [1], Wenli Li [1, 2, 3*]

[1] Key Laboratory of Marine Drugs, Ministry of Education of China, School of Medicine and Pharmacy, Ocean University of China, Qingdao 266003, China; lihongcheng@stu.ouc.edu.cn (H.L.); sjp0625@126.com (J. S.); xingkaishuai566@126.com (K. X.); meixinshi126@163.com (M. S.); xiaofei3450@ouc.edu.cn (F. X.)

[2] Laboratory for Marine Drugs and Bioproducts, Qingdao Marine Science and Technology Center, Qingdao 266237, China

[3] State Key Laboratory for Crop Stress Resistance and High-Efficiency Production, Shaanxi Key Laboratory of Natural Products & Chemical Biology, College of Chemistry & Pharmacy, Northwest A&F University, Yangling 712100, China; lizhiting@nwafu.edu.cn (Z.L.); yangxy@qdhhc.edu.cn (X. Y.)

* Correspondence: liwenli@nwafu.edu.cn (W. L.)

Table S1. ITS sequence of *Penicillium javanicum* OUCF108

CCTTGGTCAAATTAGAGGAAGTAAAAGTCGTAACAAGGTTTCCGTAGGTGAACCTG<br>CGGAAGGATCATTACCGAGTGAGGGCCCTCTGGGTCCAACCTCCCACCCGTGTTTA<br>TCGTACCTTGTTGCTTCGGCGGGCCCGCCGTCATGGCCGCCGGGGGGCACCCGCCC<br>CCGGGCCCGCGCCCGCCGAAGACACCATTGAACTCTGTCTGAAGATTGCAGTCTGA<br>GTGATTAGCTAAATCAGTTAAAACTTTCAACAACGGATCTCTTGGTTCCGGCATCG<br>ATGAAGAACGCAGCGAAATGCGATAAGTAATGTGAATTGCAGAATTCAGTGAATC<br>ATCGAGTCTTTGAACGCACATTGCGCCCCCTGGTATTCCGGGGGGCATGCCTGTCC<br>GAGCGTCATTGCTGCCCTCAAGCACGGCTTGTGTGTTGGGCCCCCGCCCCCCGGTC<br>CCGGGGGGCGGGCCCGAAAGGCAGCGGCGGCACCGCGTCCGGTCCTCGAGCGTAT<br>GGGGCTTCGTCACCCGCTCTGTAGGCCCGGCCGGCGCCCGCCGGCGACTCCCATCA<br>ATCTTTCCAGGTTGACCTCGGATCAGGTAGGGATACCCGCTGAACTTAAGCATATC<br>AAAAGGCGGAGGAA

Table S2. Functional annotation of genes in *pig1* gene cluster

Locus tag	gene	Putative function	Homolog	Protein identity (%)
g1207	*pigB1*	Glycosyltransferase	P33300.1	54%
g1208	*pigC1*	-	-	-
g1209	*pigD1*	Imidazoleglycerol-phosphate dehydratase	Q9HEG3.1	60%
g1210	*pigE1*	-	-	-
g1211	*pigF1*	-	-	-
g1212	*pigG1*	Monooxygenase	Q4WAZ2.1	32%
g1213	*pigA1*	Polyketide synthase	A0A1W7M1U5.1	48%
g1214	*pigH1*	*O*-methyltransferase	A0A443HJY8.1	46%
g1215	*pigI1*	-	-	-
g1216	*pigJ1*	-	-	-
g1217	*pigK1*	Oxidoreductase	A0A067XMP1.1	33%
g1218	*pigL1*	Flavin-binding monooxygenase	I1RF61.1	49%
g1219	*pigM1*	-	-	-
g1220	*pigN1*	-	-	-
g1221	*pigO1*	Multicopper oxidase	I1RF62.1	62%

Table S3. Functional annotation of genes in *pig2* gene cluster

Locus tag	gene	Putative function	Homolog	Protein identity (%)
g11235	*pigB2*	-	-	-
g11236	*pigC2*	Glycosyl hydrolase	P48845.1	67%
g11237	*pigD2*	DUF2422	P87136.1	26%
g11238	*pigE2*	-	-	-
g11239	*pigF2*	Multicopper oxidase	I1RF62.1	60%
g11240	*pigG2*	Fasciclin domain	I1RF63.1	43%
g11241	*pigH2*	Short chain dehydrogenase	A0A443HJZ3.1	70%
g11242	*pigA2*	Polyketide synthase	P9WET9.1	46%
g11243	*pigI2*	Flavin-binding monooxygenase	I1RF61.1	56%
g11244	*pigJ2*	MFS transporter	I1RF56.1	54%
g11245	*pigK2*	-	-	-
g11246	*pigL2*	Fungal specific transcription factor	I1RF57.1	39%
g11247	*pigM2*	Serine hydrolase	S0DRW4.1	28%
g11248	*pigN2*	*O*-methyltransferase	A0A443HJY8.1	52%
g11249	*pigO2*	Fungal specific transcription factor	I1RF54.1	25%

Table S4. The list of primers used in this study and their main characteristics

Primer	Sequence（5′→3′）
ITS1-F	CTTGGTCATTTAGAGGAAGTAA
ITS4-R	TCCTCCGCTTATTGATATGC
U-Pjku70-F	CTTGAACGTACAAATAACCT
U-Pjku70-R	GGGGCTTTTCCTTCTCTAGACACGTTCGCCATGGAGACGT
D-Pjku70-F	ACTGACTTGTGCGCTGCAATCGTAGCTCTTCAATGGCATT
D-Pjku70-R	TTCGGTCAAAGTCAATAAGT
Ppgk-1430-F	TCTAGAGAAGGAAAAGCCCC
Tpgk-1430-R	ATTGCAGCGCACAAGTCAGT
pTAex3-pigA1-F1	CTGAACAATAAACCCCACAGCAAGCTCCGAATTCATGTCC CTGAGAGCTACCGA
pTAex3-pigA1-R1	CTGGGCTGCCTGGACCAAATCAT
pTAex3-pigA1-F2	GTGCAACCCATCCTAGATGA
pTAex3-pigA1-R2	CCACCCTTCACGAGCTACTACAGATCCCCGGGTACCTCAAA GCGACCGATAAATGA
pTAex3-pigB1-F	CTGAACAATAAACCCCACAGCAAGCTCCGAATTCATGCGG CGCGGTCTCCTCATAT
pTAex3-pigB1-R	CCACCCTTCACGAGCTACTACAGATCCCCGGGTACCAAAG GCAAATCAGTCGTCGC
pTAex3-pigG1-F	CTGAACAATAAACCCCACAGCAAGCTCCGAATTCATGCGC TTCCTCTGCCTCCA
pTAex3-pigG1-R	CCACCCTTCACGAGCTACTACAGATCCCCGGGTACCCTACG CTAACGCGCAGAACC
pTAex3-pigJ1-F	CTGAACAATAAACCCCACAGCAAGCTCCGAATTCATGCGC TTCCTCTGCCTCCA
pTAex3-pigJ1-R	CCACCCTTCACGAGCTACTACAGATCCCCGGGTACCCTACG CTAACGCGCAGAACC
pTAex3-pigK1-F	CTGAACAATAAACCCCACAGCAAGCTCCGAATTCATGAAT GAAACTCCTG
pTAex3-pigK1-R	CCACCCTTCACGAGCTACTACAGATCCCCGGGTACCCTATG TCCGACGAGAATCTA
pTAex3-pigL1-F	CTGAACAATAAACCCCACAGCAAGCTCCGAATTCATGGCT GAACAAAATCCCAC
pTAex3-pigL1-R	CCACCCTTCACGAGCTACTACAGATCCCCGGGTACCCTACC CCTGCAGGCCATA
pTAex3-pigO1-F	CTGAACAATAAACCCCACAGCAAGCTCCGAATTCATGACC TCCTTCGAAAAGGC

pTAex3-pigO1-R	CCACCCTTCACGAGCTACTACAGATCCCCGGGTACCTCAAT ACGACTCAACCATATCC
pUSA-pigG1-F	AACCCCACAGCAAGCTCCGAATTCGAGCTCGGTACCATGC TCAACTATCTGTACC
pUSA-pigG1-R	ACCCTTCACGAGCTACTACAGATCCCCGGGTACCTTAGATA GTAAGGCAGGGGG
pUSA-pigJ1-F	AACCCCACAGCAAGCTCCGAATTCGAGCTCGGTACCATGC GCTTCCTCTGCCTCCA
pUSA-pigJ1-R	ACCCTTCACGAGCTACTACAGATCCCCGGGTACCCTACGCT AACGCGCAGAACCA
InF-pUSA-BamHI-F	CGCCAAGCACGCTAGTCTATTATAGGAAAGGATCCCCATC ATGGTGTTTTGATC
InF-pUSA-BamHI-R	TTGGATTTTTATATCCAAGATGACTCTAGAGGATCGTAAGA TACATGAGCTTCGG
InF-pUSA-linker-F	GCTCGCGAGCGCGTTCCACTGCATCATCAGTCTAG
InF-pUSA-linker-R	AACGCGCTCGCGAGCAAGTACCATACAGTACCGCG
InF-pAdeA-SpeI-F	GCATGCCTGCAGGTCGACTCTAGAGGATCTACTAGTCAAG AGCAGAATGTGAACG
InF-pAdeA-SpeI-R	ACTCGTATGCTGTTGCGCAGAATCCATATGACTAGTGATAC ATGAGCTTCGGTG
InF-pAdeA-linker-F	GCTCGCGAGCGCGTTCCACTGCATCATCAGTCTAG
InF-pAdeA-linker-R	AACGCGCTCGCGAGCAAGTACCATACAGTACCGCG
InF-pAdeA-XbaI-F	ATAGGGAAAGCTTGCATGCCTGCAGGTCGACTCTAGACCC ATCATGGTGTTTTGATC
InF-pAdeA-XbaI-R	AAATGATCAAAACACCATGATGGGGATCCTCTAGAGTAAG ATACATGAGCTTCGG
InF-pPTRI-SmaI-F	CATGCCTGCAGGTCGACTCTAGAGGATCCCCCCATCATGGT GTTTTGATCA
InF-pPTRI-SmaI-R	CGACGGCCAGTGAATTCGAGCTCGGTACCCGTAAGATACA TGAGCTTCGG
InF-pPTRI-linker-F	GCTCGCGAGCGCGTTCCACTGCATCATCAGTCTAG
InF-pPTRI-linker-R	AACGCGCTCGCGAGCAAGTACCATACAGTACCGCG
pTAex3-pigA2-F1	CTGAACAATAAACCCCACAGCAAGCTCCGAATTCCATGGC GAATCAACTTCAAA
pTAex3-pigA2-R1	CCCTTAGCGAGGGTCTCGAAAT
pTAex3-pigA2-F2	AGATGCTATCTTGGAAGATTTCGAG
pTAex3-pigA2-R2	CCACCCTTCACGAGCTACTACAGATCCCCGGGTACCTCATG CAAGCGCTCCAGAGA
pTAex3-pigC2-F	CTGAACAATAAACCCCACAGCAAGCTCCGAATTCATGAAA ATCTCTCTCGCGGC

pTAex3-pigC2-R	ACCCTTCACGAGCTACTACAGATCCCCGGGTACCTCACTCA ATAGACCACTGGC
pTAex3-pigF2-F	CTGAACAATAAACCCCACAGCAAGCTCCGAATTCATGCAT CTGCCCTCGTATTG
pTAex3-pigF2-R	CCACCCTTCACGAGCTACTACAGATCCTACGATATTAGATC CTCCA
pTAex3-pigG2-F	CTGAACAATAAACCCCACAGCAAGCTCCGAATTCATGGAG CGCAAGAGACTCAG
pTAex3-pigG2-R	CCACCCTTCACGAGCTACTACAGATCCCCGGGTACCCTACA AGACAGAGTCATCGA
pTAex3-pigH2-F	CTGAACAATAAACCCCACAGCAAGCTCCGAATTCATGGCG ATCGAGCCGAACGAA
pTAex3-pigH2-R	CCACCCTTCACGAGCTACTACAGATCCCCGGGTACCTCAAT AGGCGCTAGGGTGGT
pTAex3-pigI2-F	CTGAACAATAAACCCCACAGCAAGCTCCGAATTCATGTCT GACAAAGCGCCAAC
pTAex3-pigI2-R	CCACCCTTCACGAGCTACTACAGATCCCCGGGTACCTTACG GGTTCCAACCCTTCG
pTAex3-pigM2-F	CTGAACAATAAACCCCACAGCAAGCTCCGAATTCATGCAA GTACCAGTCAAGGT
pTAex3-pigM2-R	ACCCTTCACGAGCTACTACAGATCCCCGGGTACCCTACTTC TCCTCGCATATCT
pTAex3-pigN2-F	CTGAACAATAAACCCCACAGCAAGCTCCGAATTCACAATG GCTCCAGCACTCGT
pTAex3-pigN2-R	CCACCCTTCACGAGCTACTACAGATCCCCGGGTACCTCAAA GCTCAAACACGATTTCC
pUSA-pigH2-F	AACCCCACAGCAAGCTCCGAATTCGAGCTCGGTACCATGG CGATCGAGCCGAACGAA
pUSA-pigH2-R	ACCCTTCACGAGCTACTACAGATCCCCGGGTACCTCAATAG GCGCTAGGGTGGT
pUSA-pigN2-F	AACCCCACAGCAAGCTCCGAATTCGAGCTCGGTACCAATG GCTCCAGCACTCGTCG
pUSA-pigN2-R	ACCCTTCACGAGCTACTACAGATCCCCGGGTACCTCAAAGC TCAAACACGATTTCC

Table S5. The list of plasmids generated in this study and detailed information for their construction

Plasmid	Inserts	Primer 1	Primer 2	PCR Template	Vector
pTAex3-*pigA1*	*pigA1*-1	pTAex3-*pigA1*-F1	pTAex3-*pigA1*-R1	gDNA	pTAex3 digested with *Eco*RI and *Kpn*I
	pigA1-2	pTAex3-*pigA1*-F2	pTAex3-*pigA1*-R2	gDNA	pTAex3 digested with *Eco*RI and *Kpn*I
pTAex3-*pigB1*	*pigB1*	pTAex3-*pigB1*-F	pTAex3-*pigB1*-R	gDNA	pTAex3 digested with *Eco*RI and *Kpn*I
pTAex3-*pigD1*	*pigD1*	pTAex3-*pigD1*-F	pTAex3-*pigD1*-R	gDNA	pTAex3 digested with *Eco*RI and *Kpn*I
pTAex3-*pigG1*	*pigG1*	pTAex3-*pigG1*-F	pTAex3-*pigG1*-R	gDNA	pTAex3 digested with *Eco*RI and *Kpn*I
pTAex3-*pigJ1*	*pigJ1*	pTAex3-*pigJ1*-F	pTAex3-*pigJ1*-R	gDNA	pTAex3 digested with *Eco*RI and *Kpn*I
pTAex3-*pigK1*	*pigK1*	pTAex3-*piKJ1*-F	pTAex3-*pigK1*-R	gDNA	pTAex3 digested with *Eco*RI and *Kpn*I
pTAex3-*pigL1*	*pigL1*	pTAex3-*pigL1*-F	pTAex3-*pigL1*-R	gDNA	pTAex3 digested with *Eco*RI and *Kpn*I
pTAex3-*pigO1*	*pigO1*	pTAex3-*pigO1*-F	pTAex3-*pigO1*-R	gDNA	pTAex3 digested with *Eco*RI and *Kpn*I
pUSA-*pigG1*	*pigG1*	pUSA-*pigG1*-F	pUSA-*pigG1*-R	gDNA	pUSA digested with *Kpn*I
pUSA-*pigJ1*	*pigJ1*	pUSA-*pigJ1*-F	pUSA-*pigJ1*-R	gDNA	pUSA digested with *Kpn*I
pAdeA-*pigB1*	P_{amyB}- *pigB1*-T_{amyB}	InF-pAdeA-*Spe*I-F	InF-pAdeA-*Spe*I-R	pTAex3-*pigB1*	pAdeA digested with *Spe*I
pAdeA-*pigL1*	P_{amyB}- *pigL1*-T_{amyB}	InF-pAdeA-*Spe*I-F	InF-pAdeA-*Spe*I-R	pTAex3-*pigL1*	pAdeA digested with *Spe*I
pUSA-*pigG1*-*pigJ1*	P_{amyB}- *pigJ1*-T_{amyB}	InF-pUSA-*BamH*I-F	InF-pUSA-*BamH*I-R	pTAex3-*pigJ1*	pUSA-*pigG1* digested with *Bam*HI
pUSA-*pigG1*-*pigK1*	P_{amyB}- *pigK1*-T_{amyB}	InF-pUSA-*BamH*I-F	InF-pUSA-*BamH*I-R	pTAex3-*pigK1*	pUSA-*pigG1* digested with *Bam*HI
pUSA-*pigJ1*-*pigK1*	P_{amyB}- *pigK1*-T_{amyB}	InF-pUSA-*BamH*I-F	InF-pUSA-*BamH*I-R	pTAex3-*pigK1*	pUSA-*pigJ1* digested with *Bam*HI
pAdeA-*pigB1*-*pigL1*	P_{amyB}- *pigK1*-T_{amyB}	InF-pAdeA-*Xba*I-F	InF-pAdeA-*Xba*I-R	pTAex3-*pigL1*	pAdeA-*pigB1* digested with *Xba*I
pAdeA-*pigB1*-*pigO1*	P_{amyB}- *pigO1*-T_{amyB}	InF-pAdeA-*Xba*I-F	InF-pAdeA-*Xba*I-R	pTAex3-*pigO1*	pAdeA-*pigB1* digested with *Xba*I
pAdeA-*pigL1*-*pigO1*	P_{amyB}- *pigO1*-T_{amyB}	InF-pAdeA-*Xba*I-F	InF-pAdeA-*Xba*I-R	pTAex3-*pigO1*	pAdeA-*pigL1* digested with *Xba*I
pUSA-*pigG1*-*pigJ1*-*pigK1*	P_{amyB}- *pigJ1*-T_{amyB}	InF-pUSA-*BamH*I-F	InF-pUSA-linker-R	pTAex3-*pigJ1*	pUSA-*pigG1* digested with *Bam*HI
	P_{amyB}- *pigK1*-T_{amyB}	InF-pUSA-linker-F	InF-pUSA-*BamH*I-R	pTAex3-*pigK1*	

pAdeA-*pigB1-pigL1-pigO1*	P_{amyB}- *pigL1*-T_{amyB}	InF-pAdeA-*Xba*I-F	InF-pAdeA-linker-R	pTAex3-*pigJ1*	pAdeA-*pigB1* digested with *Xba*I
	P_{amyB}- *pigO1*-T_{amyB}	InF-pAdeA-linker-F	InF-pUSA-*BamH*I-R	pTAex3-*pigK1*	
pTAex3-*pigA2*	*pigA2*-1	pTAex3-*pigA2*-F1	pTAex3-*pigA2*-R1	gDNA	pTAex3 digested with *Eco*RI and *Kpn*I
	pigA2-2	pTAex3-*pigA2*-F2	pTAex3-*pigA2*-R2	gDNA	pTAex3 digested with *Eco*RI and *Kpn*I
pTAex3-*pigC2*	*pigC2*	pTAex3-*pigC2*-F	pTAex3-*pigC2*-R	gDNA	pTAex3 digested with *Eco*RI and *Kpn*I
pTAex3-*pigF2*	*pigF2*	pTAex3-*pigF2*-F	pTAex3-*pigF2*-R	gDNA	pTAex3 digested with *Eco*RI and *Kpn*I
pTAex3-*pigG2*	*pigG2*	pTAex3-*pigG2*-F	pTAex3-*pigG2*-R	gDNA	pTAex3 digested with *Eco*RI and *Kpn*I
pTAex3-*pigH2*	*pigH2*	pTAex3-*pigH2*-F	pTAex3-*pigH2*-R	gDNA	pTAex3 digested with *Eco*RI and *Kpn*I
pTAex3-*pigI2*	*pigI2*	pTAex3-*pigI2*-F	pTAex3-*pigI2*-R	gDNA	pTAex3 digested with *Eco*RI and *Kpn*I
pTAex3-*pigM2*	*pigM2*	pTAex3-*pigM2*-F	pTAex3-*pigM2*-R	gDNA	pTAex3 digested with *Eco*RI and *Kpn*I
pTAex3-*pigN2*	*pigN2*	pTAex3-*pigN2*-F	pTAex3-*pigN2*-R	gDNA	pTAex3 digested with *Eco*RI and *Kpn*I
pUSA-*pigH2*	*pigH2*	pUSA-*pigH2*-F	pUSA-*pigH2*-R	gDNA	pUSA digested with *Kpn*I
pUSA-*pigN2*	*pigN2*	pUSA-*pigN2*-F	pUSA-*pigN2*-R	gDNA	pUSA digested with *Kpn*I
pAdeA-*pigF2*	P_{amyB}- *pigF2*-T_{amyB}	InF-pAdeA-*Spe*I-F	InF-pAdeA-*Spe*I-R	pTAex3-*pigF2*	pAdeA digested with *Spe*I
pAdeA-*pigG2*	P_{amyB}- *pigG2*-T_{amyB}	InF-pAdeA-*Spe*I-F	InF-pAdeA-*Spe*I-R	pTAex3-*pigG2*	pAdeA digested with *Spe*I
pPTRI-*pigI2*	P_{amyB}- *pigI2*-T_{amyB}	InF-pPTRI-*Spe*I-F	InF-pPTRI-*Spe*I-R	pTAex3-*pigI2*	pPTRI digested with *Spe*I
pPTRI-*pigM2*	P_{amyB}- *pigM2*-T_{amyB}	InF-pPTRI-*Spe*I-F	InF-pPTRI-*Spe*I-R	pTAex3-*pigM2*	pPTRI digested with *Spe*I
pUSA-*pigH2-pigM2*	P_{amyB}- *pigM2*-T_{amyB}	InF-pUSA-*BamH*I-F	InF-pUSA-*BamH*I-R	pTAex3-*pigM2*	pUSA-*pigH2* digested with *Bam*HI
pUSA-*pigH2-pigN2*	P_{amyB}- *pigN2*-T_{amyB}	InF-pUSA-*BamH*I-F	InF-pUSA-*BamH*I-R	pTAex3-*pigN2*	pUSA-*pigH2* digested with *Bam*HI
pUSA-*pigM2-pigN2*	P_{amyB}- *pigN2*-T_{amyB}	InF-pUSA-*BamH*I-F	InF-pUSA-*BamH*I-R	pTAex3-*pigN2*	pUSA-*pigM2* digested with *Bam*HI
pAdeA-*pigF2-pigG2*	P_{amyB}- *pigG2*-T_{amyB}	InF-pAdeA-*Xba*I-F	InF-pAdeA-*Xba*I-R	pTAex3-*pigG2*	pAdeA-*pigF2* digested with *Xba*I
pAdeA-*pigF2-pigG2-pigI2*	P_{amyB}- *pigG2*-T_{amyB}	InF-pAdeA-*Xba*I-F	InF-pAdeA-linker-R	pTAex3-*pigG1*	pAdeA-*pigF2* digested with *Xba*I
	P_{amyB}- *pigI2*-T_{amyB}	InF-pAdeA-linker-F	InF-pUSA-*BamH*I-R	pTAex3-*pigI1*	

pPTRI-*pigI2*-*pigM2*	P_{amyB}- *pigI2*-T_{amyB}	InF-pPTRI-*Spe*I-F	InF-pPTRI-linker-R	pTAex3-*pigI2*	pPTRI digested with *Spe*I
	P_{amyB}- *pigM2*-T_{amyB}	InF-pPTRI-linker-F	InF-pPTRI-*Spe*I-R	pTAex3-*pigM2*	pPTRI digested with *Spe*I

Table S6. *Aspergillus oryzae* transformants constructed in this study

**Strain**	Host Strain	Plasmids used for transformation
AO-*pigA1*	*A. oryzae* NSAR1	pTAex3-*pigA1*
AO-*pigA1G1*	AO-*pigA1*	pUSA-*pigG1*
AO-*pigA1G1J1*	AO-*pigA1*	pUSA-*pigG1J1*
AO-*pigA1G1J1K1*	AO-*pigA1*	pUSA-*pigG1J1K1*
AO-*pigA1G1J1K1B1*	AO-*pigA1G1J1K1*	pAdeA-*pigB1*
AO-*pigA1G1J1K1B1L1*	AO-*pigA1G1J1K1*	pAdeA-*pigB1L1*
AO-*pigA1G1J1K1B1L1O1*	AO-*pigA1G1J1K1*	pAdeA-*pigB1L1K1*
AO-*pigA2*	*A. oryzae* NSAR1	pTAex3-*pigA2*
AO-*pigA2H2*	AO-*pigA2*	pUSA-*pigH2*
AO-*pigA2N2*	AO-*pigA2*	pUSA-*pigN2*
AO-*pigA2H2N2*	AO-*pigA2*	pUSA-*pigH2N2*
AO-*pigA2H2N2C2*	AO-*pigA2H2N2*	pAdeA-*pigC2*
AO-*pigA2H2N2I2*	AO-*pigA2H2N2*	pAdeA-*pigI2*
AO-*pigA2H2N2M2*	AO-*pigA2H2N2*	pAdeA-*pigM2*
AO-*pigA2H2N2F2*	AO-*pigA2H2N2*	pAdeA-*pigF2*
AO-*pigA2H2N2F2G2*	AO-*pigA2H2N2*	pAdeA-*pigF2G2*
AO-*pigA2H2N2F2G2C2*	AO-*pigA2H2N2*	pAdeA-*pigF2G2C2*
AO-*pigA2H2N2F2G2C2I2*	AO-*pigA2H2N2F2G2*	pPTRI-*pigI2*
AO-*pigA2H2N2F2G2C2M2*	AO-*pigA2H2N2F2G2*	pPTRI-*pigM2*
AO-*pigA2H2N2F2G2C2I2M2*	AO-*pigA2H2N2F2G2*	pPTRI-*pigI2M2*

Table S7. Minimum inhibitory concentrations (MICs) of compounds **1–5** against selected pathogenic microorganisms.

	**1**	**2**	**3**	**4**	**5**	**Tet.**	**AmB**
**Gram-positive bacterium**							
S. aureus CCARM 3090	-	12.5	-	-	-	6.25	-
E. faecalis CCARM 5172	-	-	-	-	-	>25	-
E. faecium CCARM 5203	-	-	-	-	-	0.02	-
M. luteus ML01	-	-	-	-	-	12.5	-
**Gram-negative bacterium**							
E. coli CCARM 1009	-	-	-	-	-	0.39	-
S. typhimurium CCARM 8250	-	-	-	-	-	>25	-
A. baumannii ATCC 19606	-	-	-	-	-	>25	-
K. pneumoniae ATCC 13883	-	-	-	-	-	12.5	-
P. aeruginosa 15690	-	-	-	-	-	6.25	-
**Fungi**							
C. albicans CMCC(F) 98001	-	-	-	-	-	-	0.2
C. auris	-	-	-	12.5	-	-	0.1

Notes: MIC values are expressed as $\mu g \cdot mL^{-1}$; Tet. = Tetracycline (positive control for antibacterial activity), AmB = Amphotericin B (positive control for antifungal activity); "-" indicates MIC > 50 $\mu g \cdot mL^{-1}$.

Figure S1. Schematic diagram illustrating the construction of the *Pjku70* gene knockout cassette via Fusion PCR and the homologous recombination event leading to *Pjku70* deletion in the genome. The *Pjku70* coding region (magenta) was replaced by the *ble* resistance gene (blue) through homologous recombination with the upstream (*Pjku70*-up) and downstream (*Pjku70*-down) homology arms. The sizes of the knockout cassette (3.6 kb) and the original *Pjku70* genomic locus (4.6 kb) are indicated.

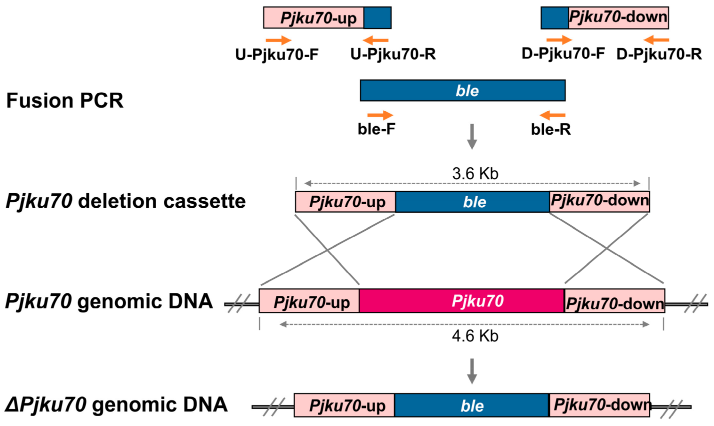

Figure S2. Schematic diagram showing the construction of the *pigA2* gene knockout cassette *via* Fusion PCR and the homologous recombination-mediated deletion of *pigA2* in the genome. The *pigA2* coding region (red) was replaced by the *neo* resistance gene (teal) through homologous recombination with the upstream (*pigA2*-up) and downstream (*pigA2*-down) homology arms. The sizes of the *pigA2* knockout cassette (3.6 kb) and the native *pigA2* genomic locus (4.6 kb) are indicated.

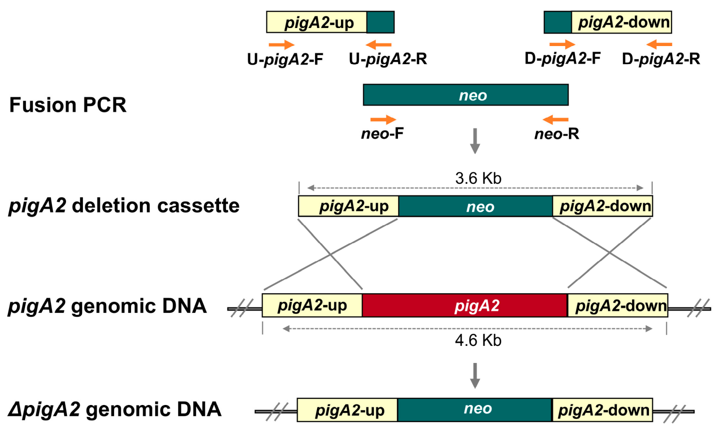

Figure S3. (A) Schematic diagram of the pET28a-PigN2 recombinant expression vector, which harbors the *pigN2* gene fused with a 6×His tag, along with key elements including the T7 promoter (T7), lac repressor gene (*lacI*), kanamycin resistance cassette (Kan^R), and origin of replication (*ori*). (B) SDS-PAGE analysis of the affinity-purified PigN2 protein; the molecular weight of the target protein is 49.0 kDa, with M representing the protein molecular weight marker.

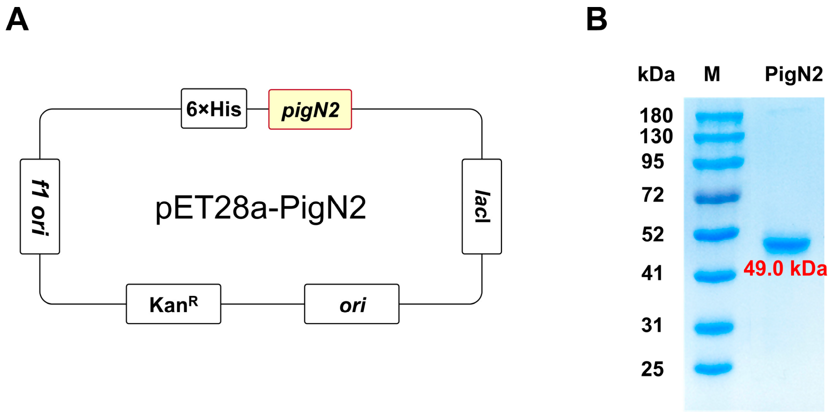

Figure S4. HPLC analysis (λ=280 nm) of the catalytic activity of PigN2 towards diverse substrates, including 1,8-dihydroxynaphthalene (DHN), YWA3, YWA2, and emodin. Chromatograms show the retention profiles of substrates alone and in the presence of PigN2; chemical structures of substrates and their corresponding methylated products (rubrofusarin from YWA2, physcion from emodin) are depicted, with no detectable product formation for DHN and YWA3 upon incubation with PigN2.

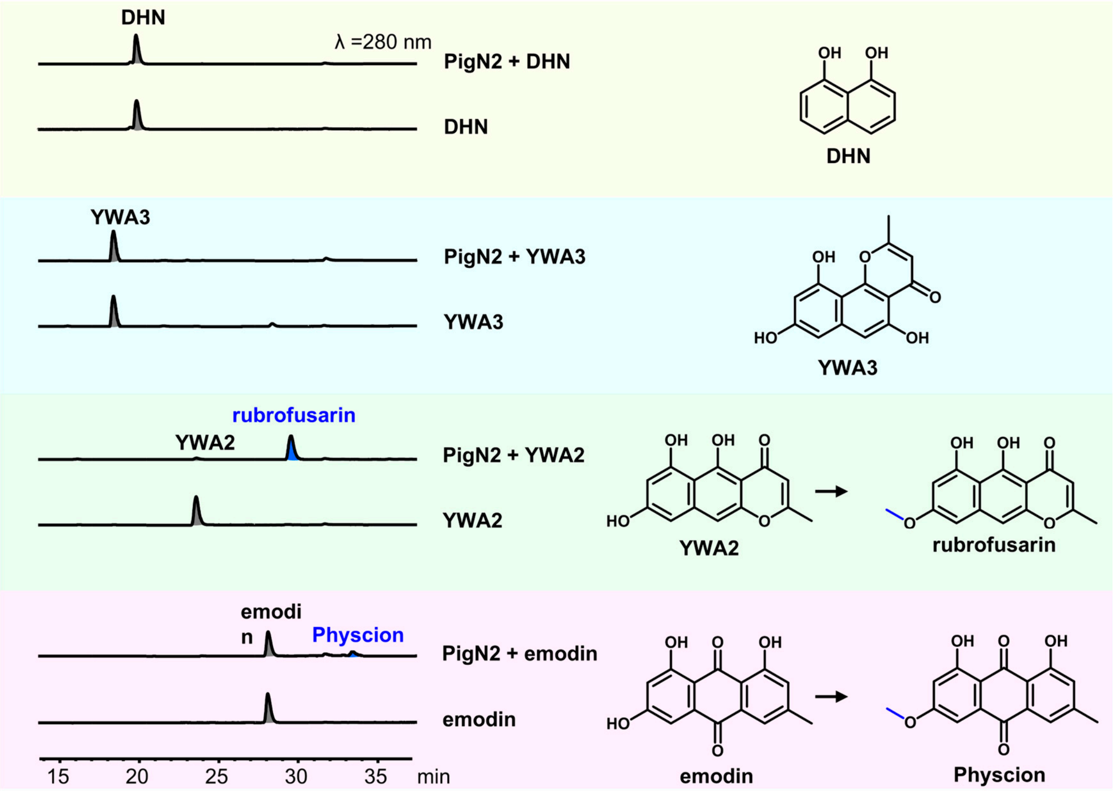

Figure S5. The UV absorption of compound **4**

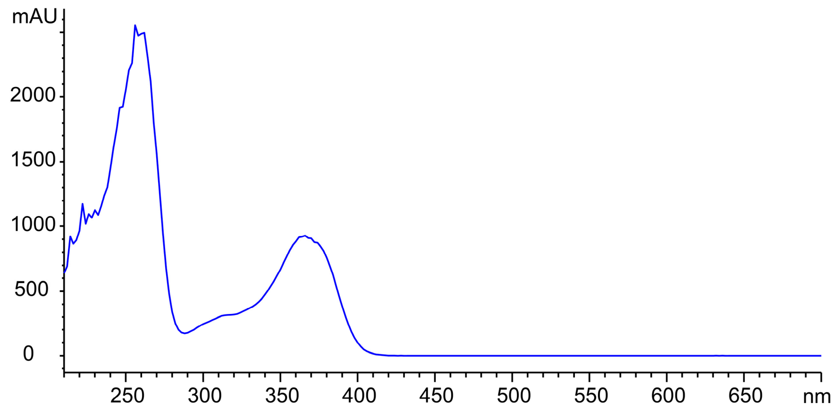

Figure S6. The HRESIMS spectrum of compound **4**

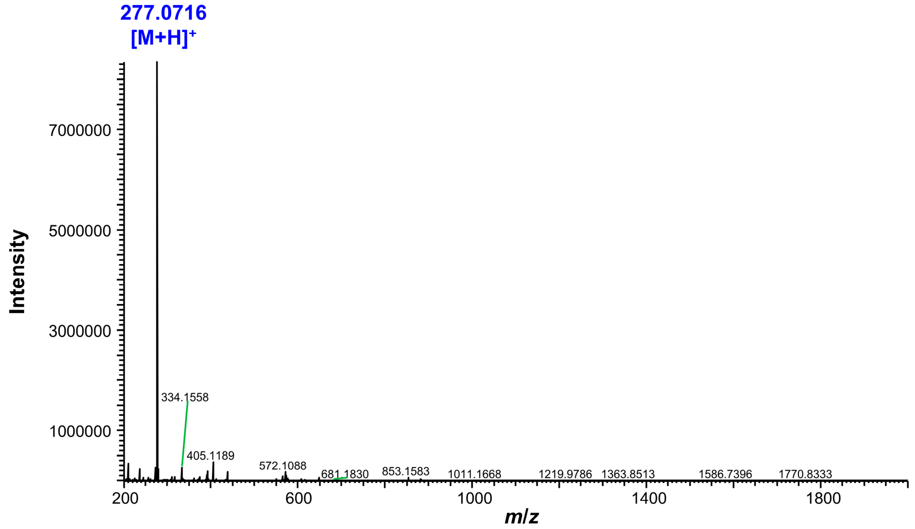

Figure S7. The ^{1}H NMR spectrum of compound **4** in DMSO-d_6 (400 MHz)

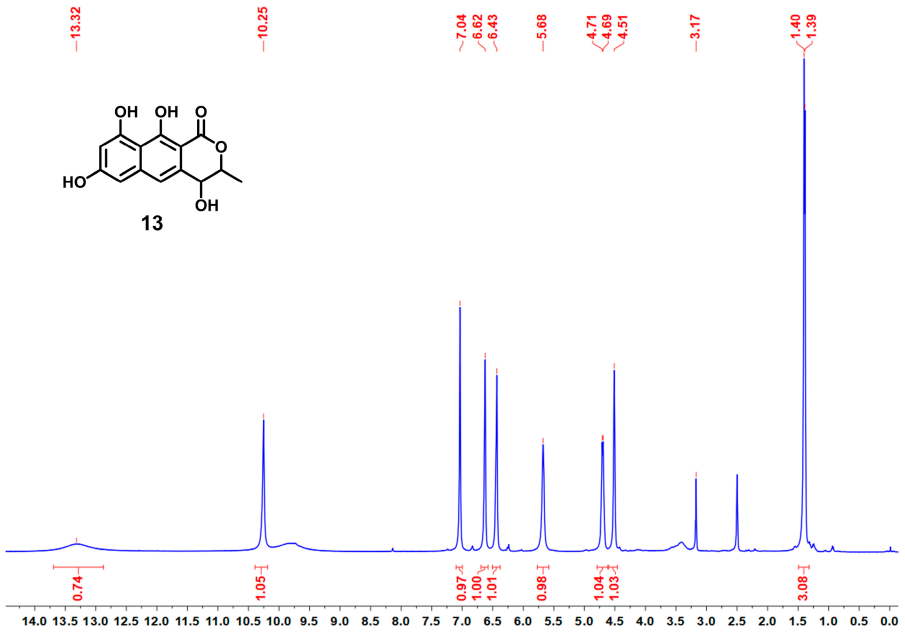

Figure S8. The ^{13}C NMR spectrum of compound **4** in DMSO-d_6 (125 MHz)

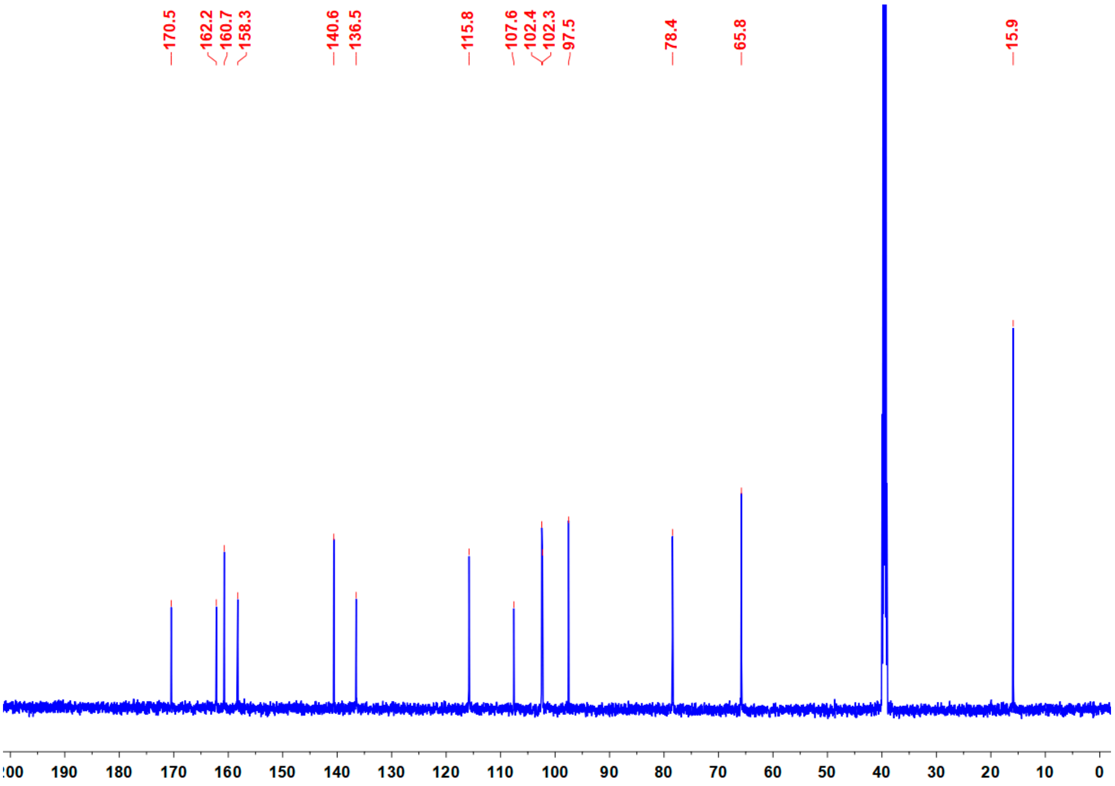

Figure S9. The HSQC spectrum of compound **4**

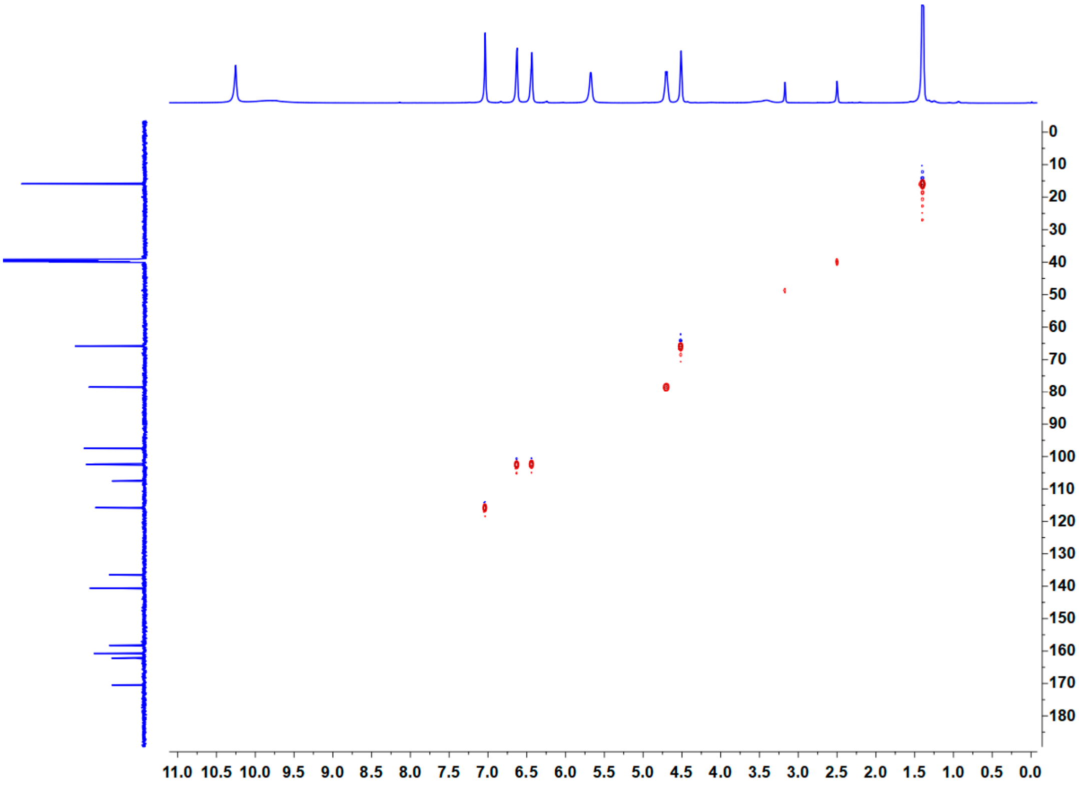

Figure S10. The ^{1}H-^{1}H COSY spectrum of compound **4**

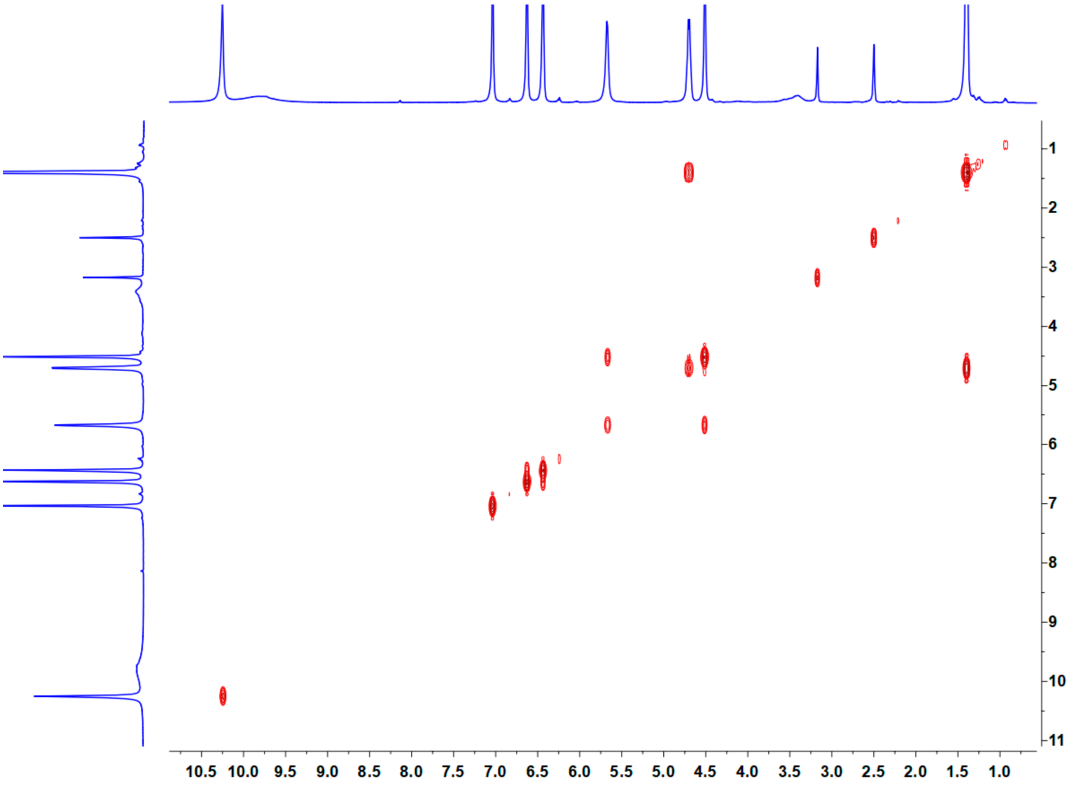

Figure S11. The HMBC spectrum of compound **4**

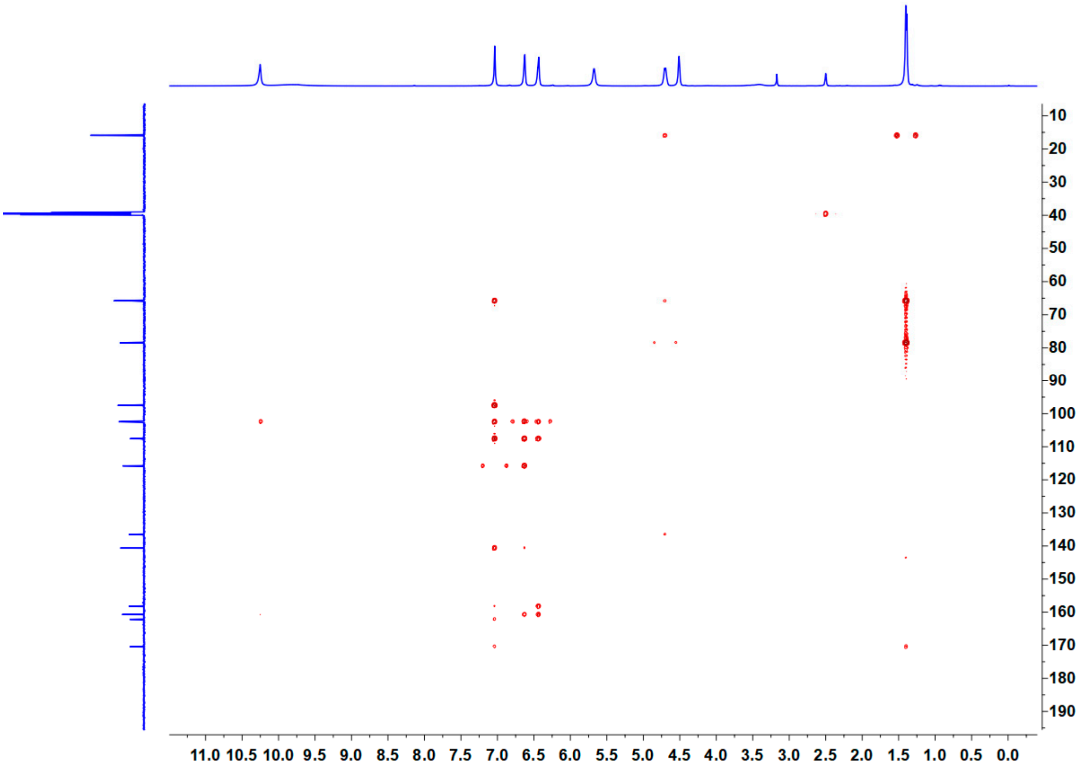

Figure S12. Comparison of the experimental CD spectrum of compound **4** with the TDDFT-calculated ECD spectra of the (4*S*) and (4*R*) isomers.

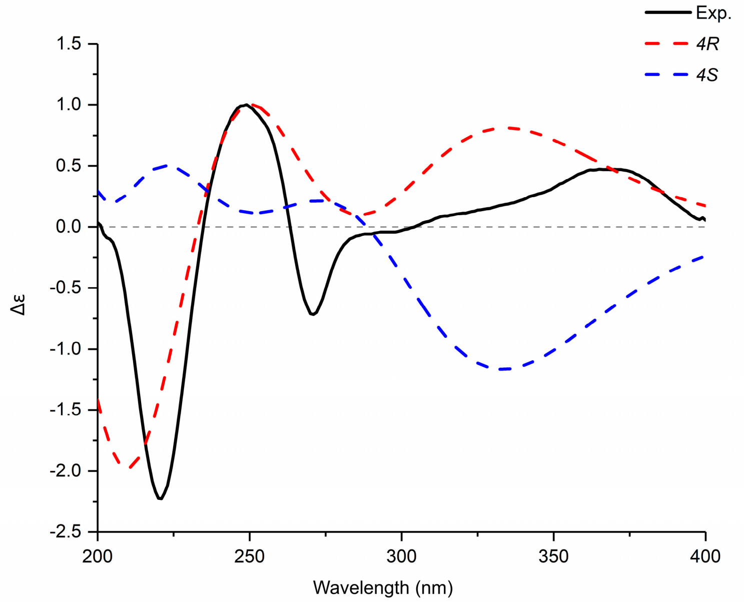

Figure S13. The UV absorption of compound **6**

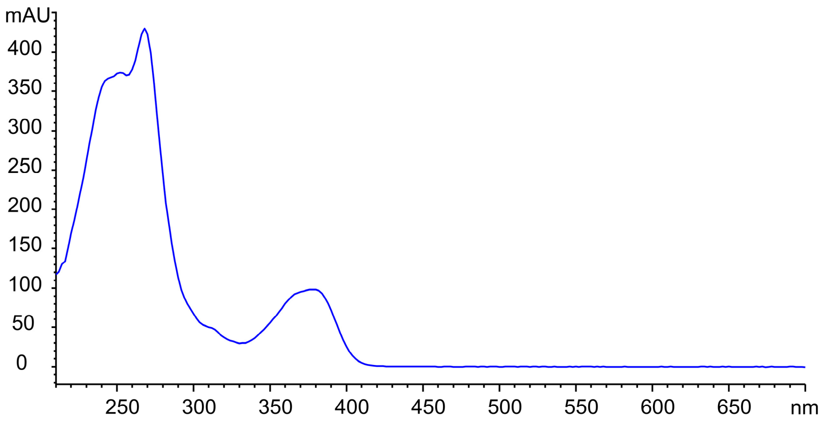

Figure S14. The HRESIMS spectrum of compound **6**

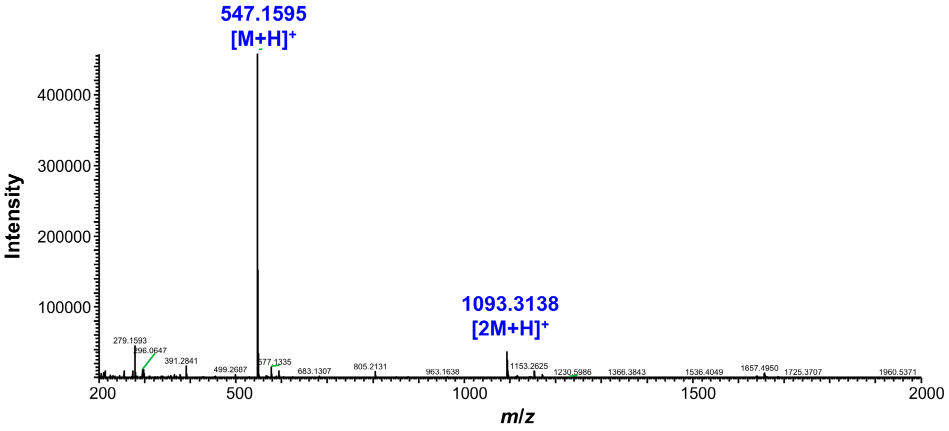

Figure S15. The UV absorption of compound **7**

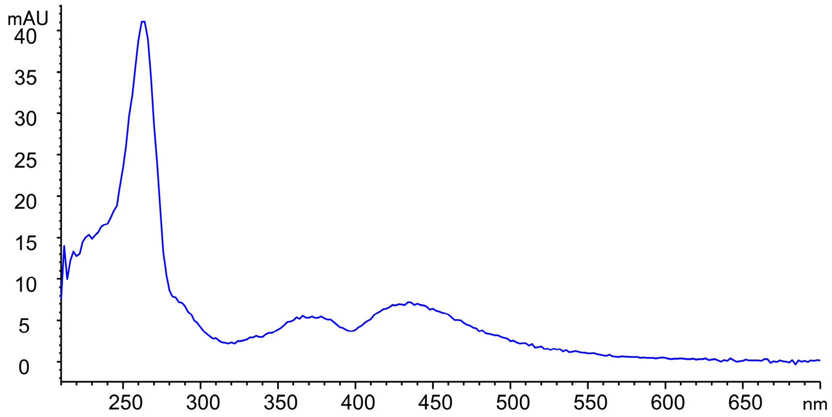

Figure S16. The HRESIMS spectrum of compound **7**

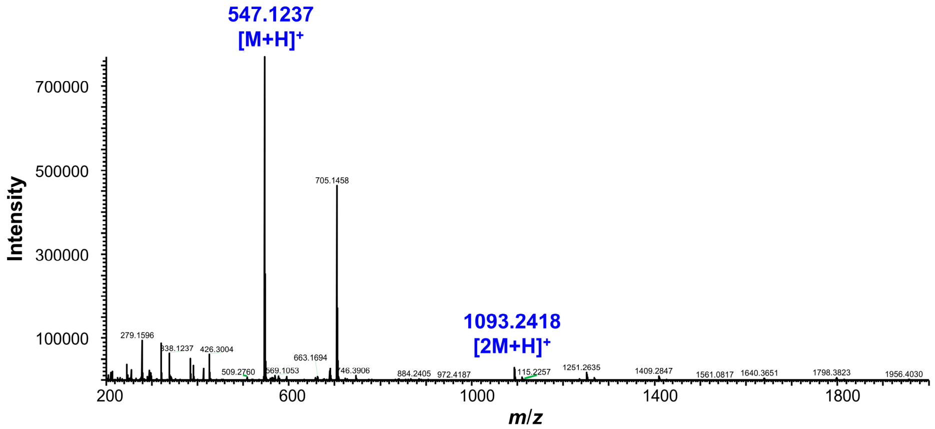

Figure S17. The UV absorption of compound **8**

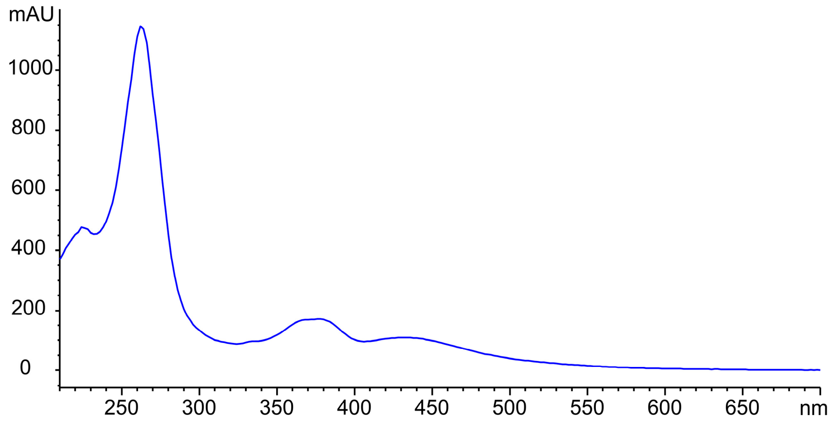

Figure S18. The HRESIMS spectrum of compound **7**

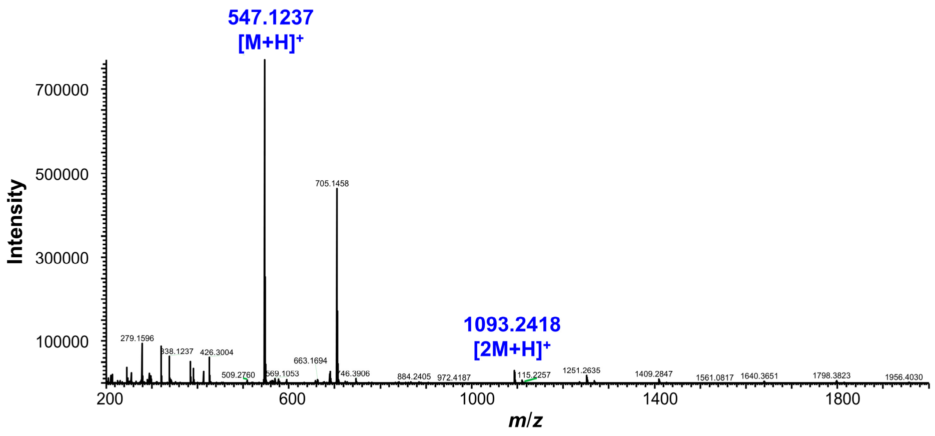

Figure S19. The UV absorption of compound **9**

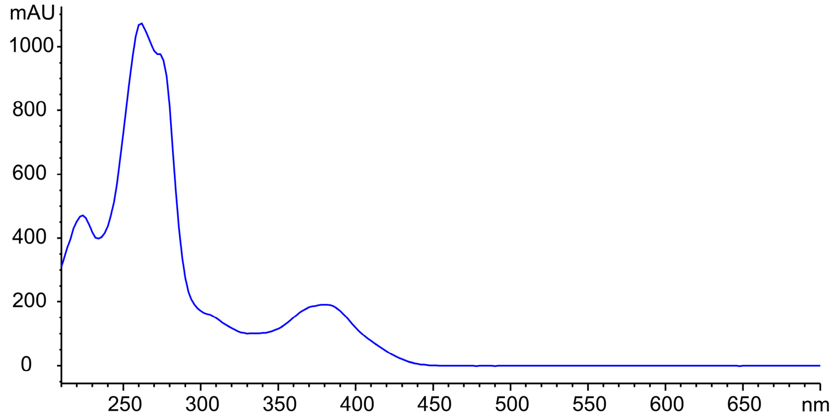

Figure S20. The HRESIMS spectrum of compound **9**

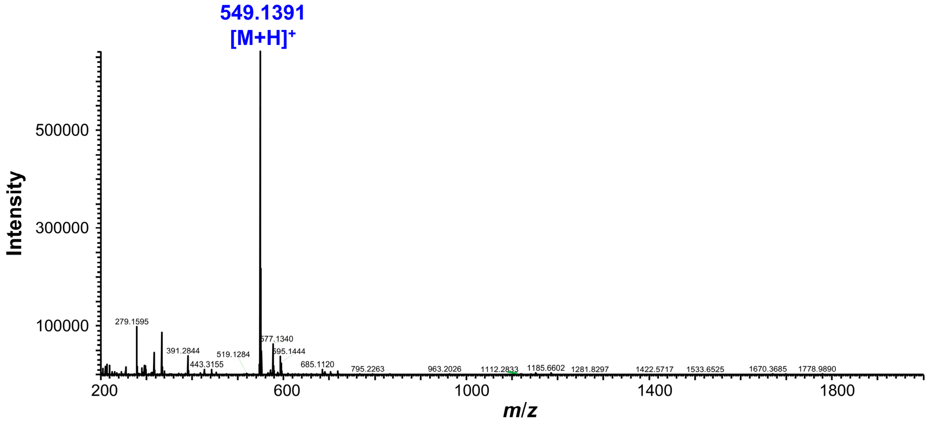

Figure S21. The UV absorption of compound **10**

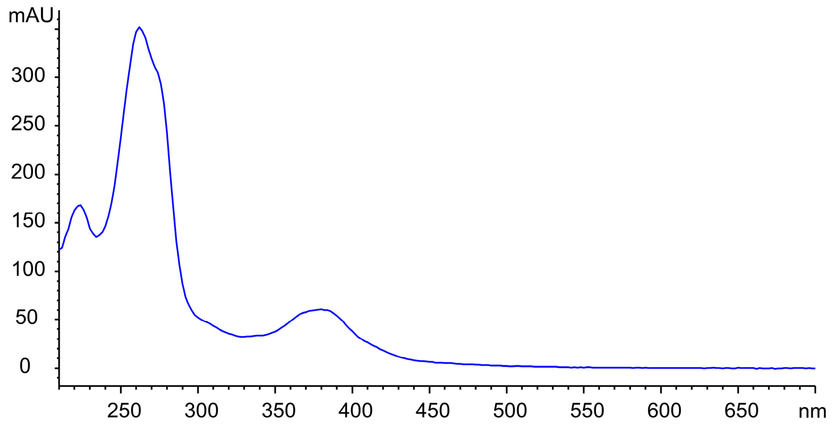

Figure S22. The HRESIMS spectrum of compound **10**

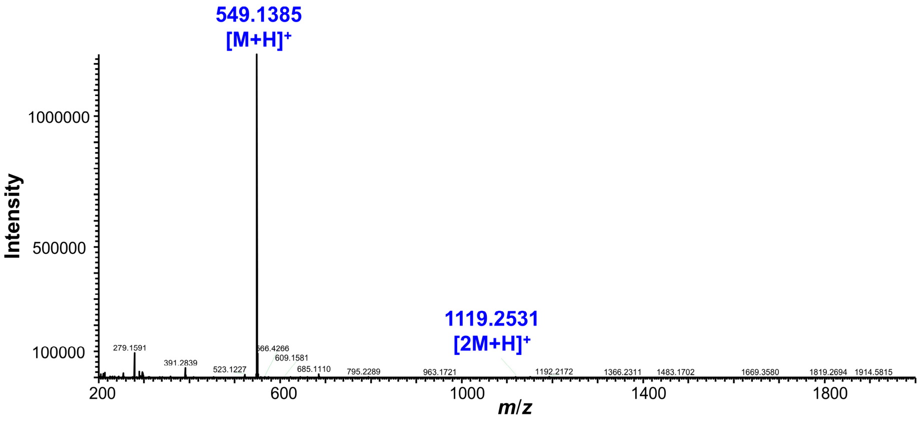

Supplement: Supplementary file 1 [file ijms-27-04156-s001.zip › ijms-4291021-supplementary.pdf]
